# Running Assessed Maximal Oxygen Uptake Increases with Participant Classification Framework Tier in Overall Cross-Country Skiing Performance: A Systematic Review with Meta-regressions

**DOI:** 10.1186/s40798-026-01001-4

**Published:** 2026-06-09

**Authors:** Steven E. Morton, Laura J. Forrest, Mykolas Kavaliauskas, Chris Easton, Kurt Rumley, Tom W. Macpherson

**Affiliations:** 1 Sport and Physical Activity Research Institute, School of Health and Life Sciences, University of the West of Scotland, Glasgow, UK; 2 Institute of Life and Earth Sciences, Heriot-Watt University, Edinburgh, UK; 3 Sportscotland Institute of Sport, Stirling, UK; 4 School of Sport, Exercise and Rehabilitation, Northumbria University, Newcastle, UK

## Abstract

**Background:**

Previous research in cross-country skiing (XCS), frequently emphasises the importance of maximal oxygen uptake (VO_2Max_) and individual studies have correlated XCS performance with VO_2Max._ However, meta-regression analyses with multiple studies have not previously been conducted.

**Objective:**

The aim of this study was to conduct meta-analyses of VO_2Max_ data, in relation to XCS performance using retrospective participant classification framework (PCF) scoring.

**Methods:**

Electronic databases were searched, up to November 2024, using Preferred Reporting Items for Systematic reviews and Meta-Analyses guidelines. Data were extracted from the included studies (n = 78), with participant groups separated by exercise mode and sex, and retrospectively scored using the PCF. Random effects meta-regressions with sub-grouping were conducted, to calculate pooled mean values by PCF tier, standard error, and 95% confidence intervals.

**Results:**

Sufficient data were only present for inferential analysis of running based protocols. VO_2Max_ values generally increased with PCF tier in males and females for both absolute (Male: R^2^ = 0.80; Female: R^2^ = 0.66) and relative to total body mass values (Male: R^2^ = 0.41; Female: R^2^ = 0.69).

**Conclusion:**

Performance prediction is multifaceted, particularly within XCS where numerous physiological parameters are compounded by multiple skiing techniques. These findings emphasise the importance of VO_2Max_ in XCS to develop performance across the participation spectrum. Even within ‘elite’ athletes, this remains true, possibly reflecting a lack of homogeneity of VO_2Max_ values within this sub-population. The values presented within this study may represent useful benchmark values for talent identification and performance development purposes.

## Key Points

- The performance classification framework is a useful tool to address incompatibilities in prior research.
- Maximal oxygen uptake (VO_2Max_) increases across all cross-country skiing performance tiers, reinforcing the role of aerobic capacity as a critical determinant of success in XCS.
- A lack of skiing-specific data and inconsistencies in defining and measuring VO_2Max_ highlight the need for future research to refine these metrics in the context of sport-specific techniques.

## Introduction

Cross-country skiing (XCS), also known as Nordic skiing, requires individuals to complete hilly and undulating courses as fast as possible. Sprint event distances ranging between 1.2 and 1.8 km, distance events up to 50 km and classic long distance events > 50 km [1, 2]. Even during sprint events, multiple high intensity heats (approximately three minutes) with limited recovery time (as little as 15 min), demands a high level of endurance, amongst several other determining factors [3–6]. The key physiological factors influencing endurance performance have been broadly categorised into three components: ‘performance oxygen uptake’ ($\dot{V}$O_2_)/aerobic (including maximal oxygen uptake ($\dot{V}$O_2Max_)), ‘performance O_2_ deficit’/anaerobic, and gross mechanical efficiency [7]. Importantly, fractional utilisation of $\dot{V}$O_2Max_ at distinct physiological points, such as the so called ‘lactate thresholds’, across different skiing techniques, and, total buffering capacity, are also important contributory components of endurance performance that are outside the focus of this review [7]. For in depth deconstruction of the broader physiological components of XCS performance, we direct readers to several excellent previously published articles [3–6].

Among these factors, $\dot{V}$O_2Max_ is frequently studied and reported in XCS research, with athletes achieving some of the highest $\dot{V}$O_2Max_ values of any sport [1, 8–10]; and is often considered among performance predictive variables [11–15]. It is therefore a measure highly valued by athletes and coaches. This apparent predictive quality somewhat contrasts to other endurance sports, such as distance running, whereby whilst $\dot{V}$O_2Max_ is important, and *does* separate ‘good’ from ‘elite’ runners [16–20], the performance predictive quality of $\dot{V}$O_2Max_ is reduced when a group of homogenous elite runners are considered [18, 21]_._ Thus, other physiological, biomechanical, psychological and/or other external factors (e.g. race pacing tactics) become more distinguishing factors where $\dot{V}$O_2Max_ homogeneity exists [16, 20]. Given that XCS is also an endurance-based sport, it is interesting that $\dot{V}$O_2Max_ is reportedly so differentiating for performance outcomes, seemingly even at the highest levels of performance [22].

Despite the volume of $\dot{V}$O_2Max_ data, meta-analyses and meta-regressions of $\dot{V}$O_2Max_, in relation to XCS performance, have proven elusive, likely owing to various methodological challenges and incongruities [1, 3, 9]. As XCS research commonly utilises relatively small sample sizes, inferences from individual studies may be restricted by such limitations. Consequently, there is justification to collate data across studies, in order to evaluate findings across a range of performance standards with increased population sizes. Furthermore, understanding of current benchmarks for physiological variables across a range of performance levels, may provide valuable insights into the talent identification and development of XCS athletes. The recently published Participant Classification Framework (PCF) [23] presents potential opportunities to overcome some of the reported challenges concerning athletic performance definitions across differing studies. Therefore, the purpose of this review was to conduct meta-regressions of $\dot{V}$O_2Max_ data, in relation to XCS performance standards using retrospective PCF scoring.

## Methods

### Systematic Search Methodology

A systematic search of research literature was conducted up to November 2024, using the Preferred Reporting Items for Systematic Reviews and Meta-Analyses (PRISMA) statement as guidance [24]. Five academic electronic databases were searched (SPORTDiscus, PubMed, Web of Science, MEDLINE, and Scopus), using key words. Search terms encompassed appropriate ski sports combined with terms for physiological testing and performance prediction. Search terms were combined using Boolean logic, specifically keywords were combined within terms using the ‘OR’ operator and the three search terms were combined using the ‘AND’ operator. The final search phrase was "cross-country skiing" OR “cross-country skier” OR “Nordic combined skiing” OR “Nordic combined skier” OR “winter biathlon” OR “winter biathlete” AND “$\dot{V}$O_2_ Max” OR “$\dot{V}$O_2_ Peak” OR “maximal aerobic*” OR “maximal oxygen*” OR endurance* OR “graded exercise test” OR “maximal anaerobic*” OR “power” OR “critical power” OR “W’” OR “strength” OR “dynamomet*” OR “maximal accumulated oxygen deficit” OR “haematocrit” OR “haemoglobin” OR “lactate” OR “blood acid base” OR “aerobic fitness” OR “anaerobic threshold” OR “W Prime” OR “max* $\dot{V}$O2” OR “cardio* fitness” OR “body composition” OR “body-fat*” OR “maximal-speed*” OR “maximal-velocity” OR “maximal-intens*” OR “maximal-effort*” OR “time to exhaustion” OR “time-trial” OR “3 min all-out” AND predict* OR perform*.

### Inclusion and Exclusion Criteria

Studies were included if reported participants were healthy and fully abled bodied cross-country skiers, competing in sprint and/or distance events for recognised Winter Olympic events and aged between 16 and 40 years old. Studies also needed to report a $\dot{V}$O_2Max_ variable, either in relative (mL.kg.min^−1^) or absolute terms (mL.min^−1^) from a graded exercise test, sufficient information to classify participants according to the PCF and statistical measures of central tendencies. The following exclusion criteria hierarchy was used to select articles for review and analysis:

1. Full article unavailable or not in English language.
2. Non-empirical study (i.e. review, case-study, survey, opinion piece).
3. Participants outside the target age demographic (< 16 or > 40 years).
4. Investigated a specialist population (e.g. para-athletes, return from injury or illness, mental health).
5. Lacked a required $\dot{V}$O_2Max_ variable.
6. Lacked sufficient details for PCF assessment.
7. Did not separate data (e.g. males from females or winter biathletes from XCS athletes).
8. Lacked other necessary statistical detail (e.g. sample size).
9. Utilised a time-trial based assessment method for $\dot{V}$O_2Max._
10. Did not include competitors in typical Winter Olympic/World Cup XCS events.
11. Duplicate participant data already included from other studies.

### Study Screening and Selection

**Fig. 1:**
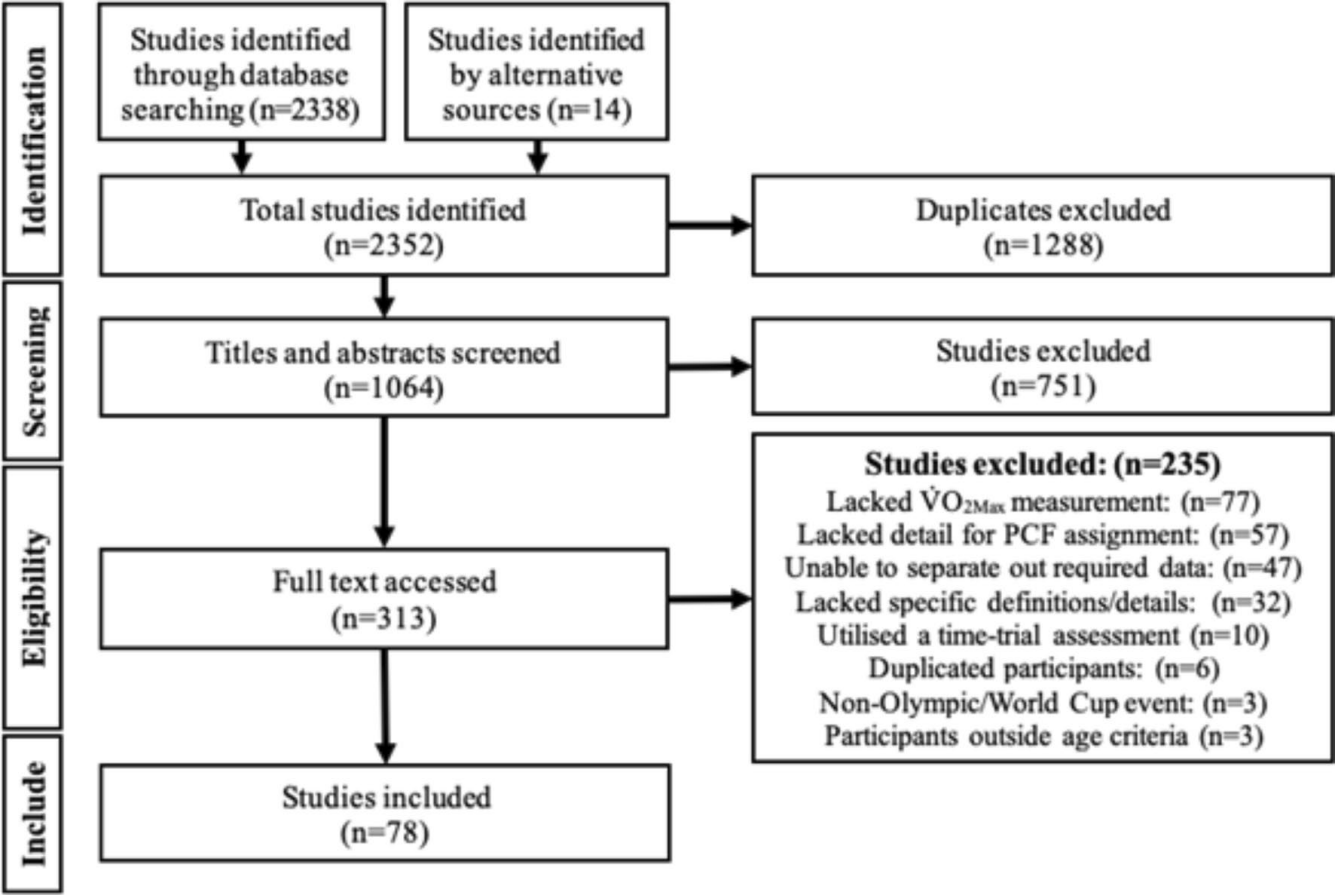
Systematic review study screening process. PCF, Participant Classification Framework; $\dot{V}$O_2Max_, Maximal Oxygen Uptake

**Fig. 2:**
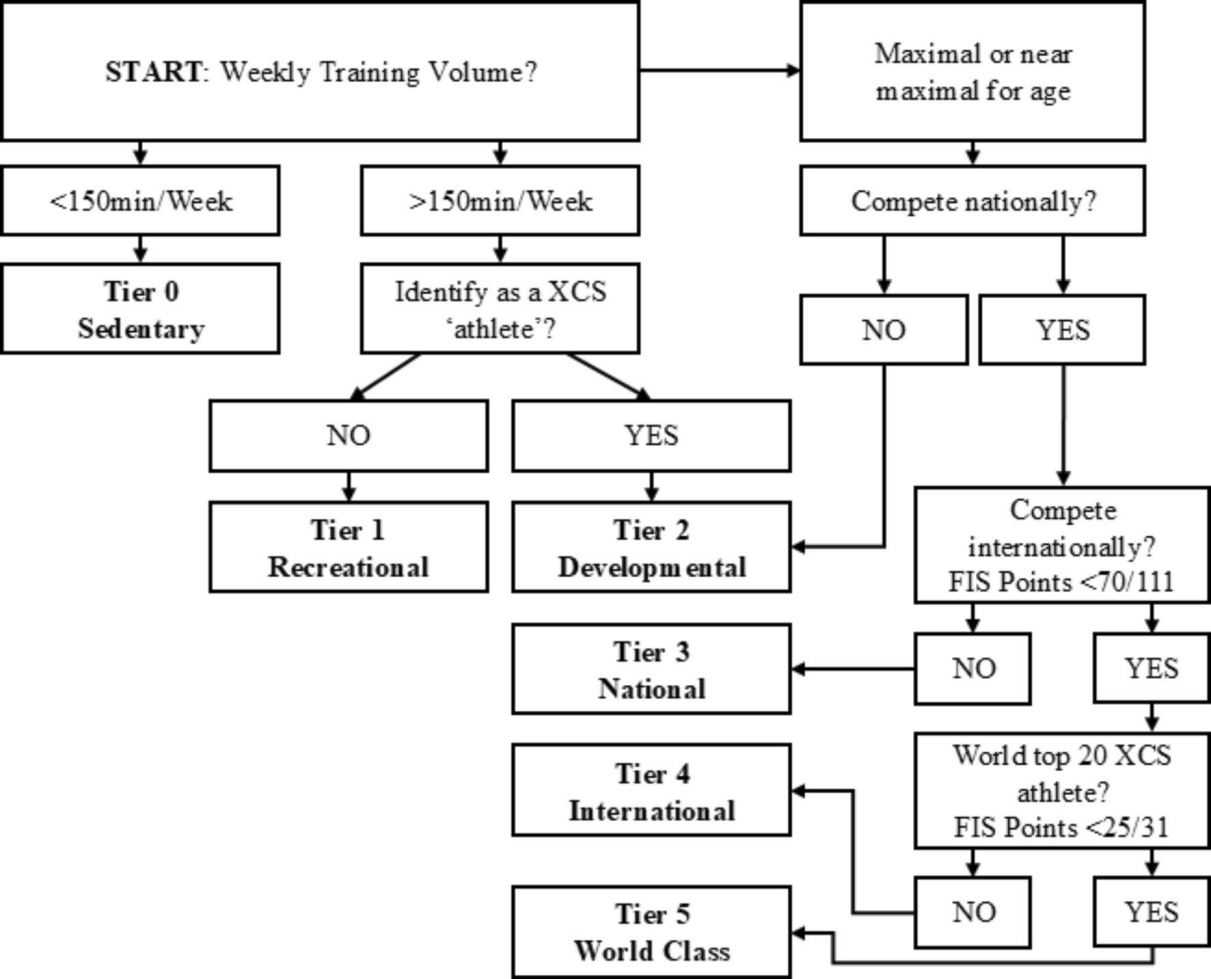
Participant classification framework decision making flow diagram. FIS, International Skiing Federation; XCS, Cross-Country Skiing

Following literature searching, studies were initially screened by titles and abstracts for relevance by two authors (SM & TM), using the defined exclusion criteria. Consensus was reached by discussion, where disagreements occurred (n = 10, at the title and abstract level) (Fig. 1). Identified texts were accessed, in full, and screened a second time for inclusion. Studies identified for data extraction were assigned a PCF tier using the PCF flow-diagram (Fig. 2) by the same two authors. Again, consensus was reached following discussion for both study inclusion and PCF scoring, where disagreements occurred (inclusion: n = 5; PCF scoring: n = 4, at the full text level).

### Performance Definitions

In undertaking this review, specific information from the original framework [23] was adjusted for application to XCS (Table 1). Where International Skiing Federation (FIS) points were reported, these were checked against a FIS point threshold, calculated using the fourth FIS season point lists (2020/2021–2023/2024 seasons). The FIS point system is a snow sport specific metric used to rank overall on-snow performance over a calendar year [25]. Individuals were removed from calculations if they had competed in less than five events, due to adjustments made to their FIS points. Remaining athletes’ data were used to produce a combined FIS point average, before sorting all records in ascending order. The individual in 20th position for males and females was identified and their averaged FIS points recorded for each year. The four annual average FIS point values were then again averaged, producing a grand mean FIS point score for 20th position. The variance for each year to the grand mean was calculated and expressed as a percentage. This variance was averaged across the four years and then added to the grand mean. Finally, the resulting number was rounded up to the nearest whole number, to account for season-to-season variability and provide leniency in the calculation. This process was completed for the tier 4/3 boundary using the individual in 300th position, as per PCF recommendations (Table 2). Participants were considered tier 5 (‘World-Class’) if reported as previously finishing in the top 20 of a World Cup, Winter Olympic or World Championship race. Participants were considered tier 4 if they were described as competing internationally or for their national team, tier 3 if they were described as ‘national level’ athletes or descriptors to this effect, tier 2 if described as ‘developmental athletes’, ‘regional competitors’ or descriptors to this effect, and tier 1 if reported as ‘recreational’ or otherwise trained but not identifying as XCS specific athletes (Fig. 2).

**Table 1:** Recommended application of a participant classification framework for cross-country skiing

Tier	Classification criteria
Tier 5: World class	Olympic/World Championship/Overall World Cup medallist
	Within the top 20 of a recently published FIS point list
	Combined FIS point total below acknowledged value for 20th position (i.e. currently suggested as being < 25 for males and < 31 for females)
	Maximal/near maximal training, typically ~ 750–950 h/year (see Holmberg [1])
	Exceptional skill-level achieved
	Olympic/World Championship/Overall World Cup medallist
Tier 4: Elite/international level	Competing at the international level (i.e. on a national team)
	Within the top 300 of a recently published FIS point list
	Combined FIS point total below acknowledged value for 300th position (i.e. currently suggested as being 25–70 for males and 31–111 for females)
	Maximal/near maximal training, typically ~ 750–950 h/year (see Holmberg [1])
	Highly proficient in skills required
Tier 3: Highly trained/national level	Competing at a recognised national level
	National Collegiate Athletic Association Division I athletes
	Completing structured and periodised training at ~ 80% typical age norms (see Karlsson et al. [26])
	Developing proficiency in skills required to perform sport
Tier 2: Trained/developmental	Competing at a local/regional level
	National Collegiate Athletic Association Division II and III athletes
	Regular training ~ 3 times/week, with purpose to compete
	Identify as a cross-country skier
	Limited skill development
Tier 1: Recreationally active	Meet World Health Organization minimum activity guidelines: Adults aged 18–64 years old completing at least 150 to 300 min moderate-intensity activity or 75–150 min of vigorous-intensity activity a week, plus muscle-strengthening activities 2 or more days a week (see Bull et al. [157])
	May participate in multiple sports/forms of activity
Tier 0: Sedentary	Do not meet minimum activity guidelines
	Occasional physical activity (e.g. walking to work, gardening or other domestic activities)

**Table 2:** Calculations for PCF Tiers 3/4/5 FIS combined point thresholds

	Combined FIS Points ($\bar{X}$ )	$\bar{X}$GΔ≅ (%)	XG + XG Δ	Threshold (Combined FIS Points)
*Males* Season 20–21 [20th]	24.3	11.3		
Season 21–22 [20th]	25.0	14.8		
Season 22–23 [20th]	20.2	7.2		
Season 23–24 [20th]	17.7	18.9		
**Grand Mean** $\bar{X}$ (G)	**21.8**	**13.1**	**24.6**	** < 25**
Season 20–21 [300th]	69.6	2.5		
Season 21–22 [300th]	65.7	3.2		
Season 22–23 [300th]	66.9	1.4		
Season 23–24 [300th]	69.3	2.1		
**Grand Mean** $\bar{X}$ (G)	**67.9**	**2.3**	**69.4**	** > 25 but < 70**
*Females* Season 20–21 [20th]	33.1	20.7		
Season 21–22 [20th]	25.3	7.8		
Season 22–23 [20th]	27.5	0.1		
Season 23–24 [20th]	23.9	13.0		
**Grand Mean** $\bar{X}$ (G)	**27.5**	**10.4**	**30.2**	** < 31**
Season 20–21 [300th]	106.6	1.4		
Season 21–22 [300th]	95.2	9.5		
Season 22–23 [300th]	105.6	0.4		
Season 23–24 [300th]	113.2	7.7		
**Grand Mean** $\bar{X}$ (G)	**105.1**	**4.7**	**110.1**	** > 31 but < 111**

*FIS*, International Skiing Federation; *PCF*, Participant Classification Framework; $\bar{X}$, Mean Average; $\bar{X}$*G*, Grand Mean Average; $\bar{X}$*G*Δ, Difference to Grand Mean. Combined FIS Points = (FISDist + FISSpr) ÷ 2

### $\dot{V}$O_2Max_ Inclusion

Reported $\dot{V}$O_2Max_ values were eligible for inclusion if reported as absolute values (L.min^−1^) or relative to whole body mass (mL.min^−1^.kg^−1^). Studies reporting $\dot{V}$O_2Peak_ with supporting primary $\dot{V}$O_2Max_ criteria (i.e., $\dot{V}$O_2_ plateau despite further increase in work) were considered to represent a mode specific $\dot{V}$O_2Max_ and were therefore included. Intervention studies including multiple assessments of $\dot{V}$O_2Max_ at different times, were combined using appropriate calculations, as described in the Cochrane guidelines, to avoid double counting [27].

### Data Extraction and Statistical Analysis

Data were extracted directly from article tables, text or by digitisation of figures (PlotDigitizer, v3.1.4, Automeris LLC, TX, USA), by a single author (SM). Axis ends and data were digitised using the closest centre point. Where overlapping data made individual datum centre points unclear, these were estimated using any visual point of reference available. Random effects meta-analyses with meta-regressions and sub-grouping were conducted using Comprehensive Meta-Analysis (v.4.0, Biostat Inc., NJ, USA). Data were separated by exercise modality or skiing technique and by participant sex. Subsequent data were sub-grouped by the attributed PCF tier. Uncertainty in the pooled effects was expressed at 95% confidence intervals [28]. A 95% prediction interval was derived to quantify the expected range of true exponents for 95% of similar future studies [29]. Between-estimate heterogeneity was quantified using the tau statistic (*τ*)—a standard deviation which describes the typical variability in the mean size exponent between studies [30]. Meta-regressions were used to explain the variance of the pooled $\dot{V}$O_2Max_ by using the *τ* and r^2^ values. Sufficient data were only available to conduct detailed sub-group regression analysis on studies which measured $\dot{V}$O_2Max_ using running based protocols.

## Results

### Search Results

A total of 2338 studies were retrieved through database searches. A further 14 studies were found through reference list scanning (2352 total studies). Of this total, 1288 studies were excluded as duplicates and 751 studies were excluded for irrelevancy after screening titles and abstracts. A total of 313 studies were accessed in full for further review, and 78 studies were deemed to meet the inclusion criteria and were selected for data extraction and analysis (Fig. 1). Key information and outcome measures for each of the included studies is summarised in Table 3.

**Table 3:** Summary of studies reporting maximal oxygen uptake as part of a study focussing on cross-country skiing. Data reported as mean ± standard deviation (SD)

Study (year)	n =	Sex (m/f)	Spr/dist/ both	Age (years)	PCF (1–5)	Participant description	$\dot{V}$O_2Max_ (mL.kg^−1^.min^−1^)	$\dot{V}$O_2Max_ (L.min^−1^)	Protocol description	Instrumentation	Calculation method
Alsobrook and Heil [31]	10	M	NR	24 ± 7	2	‘Ranged from 3 subjects among the top collegiate	57.4 ± 4.1	4.24 ± 0.49	DP on SkiErg (WB); Continuous ramp. Start: M = 40 W, F = 30 W;	Modified Concept 2 rowing ergometer;	‘Sample interval for computed
	5	F		26 ± 6	2	skiers in the USA to weekend citizen racers’	43.2 ± 4.7	2.51 ± 0.31	Power Increased: M = 20 W, F = 15 W every minute	TrueMax 2400 metabolic system	$\dot{V}$O_2_ values was 20 s’
Andersson and McGawley [32]	11	M	NR	NR^a^	2	‘Junior athletes, national and/or international standard’	67.2 ± 2.6	NR	DS on Treadmill; Continuous ramp. Start: 10.0–12.0 km.h^−1^ and 3.0° − 4.0° incline. incline increased until 9.0° (rate NR) after which, speed increased by 0.4 km.h^−1^ every minute	Rodby motorised treadmill; PRO-Ski C2 roller skis; AMIS 2001 model C metabolic system	‘Highest 30 s moving average was used to calculate $\dot{V}$O_2Max_’
	10	F		NR^a^			54.7 ± 3.5	NR			
Andersson et al. [33]	11	M	Both	24 ± 4	4	‘Swedish male cross-country competing at a national or international level’	NR	5.10 ± 0.50	DS on Treadmill; Continuous ramp. Start: 9.0 km.h^−1^ and 7.0° incline. Speed increased by 0.5 km.h^−1^ every 45 s	Rodby motorised treadmill; Pro-Ski C2 roller skis; AMIS 2001 model C metabolic system	‘Average of the three highest consecutive 10 s $\dot{V}$O_2_ values was defined as $\dot{V}$O_2Max_’
							NR	4.90 ± 0.50	DP on Treadmill; Continuous ramp Start: 21.0 km.h^−1^ and 1.0° incline. Speed increased by 1.0 km.h^−1^ every minute		
Andersson et al. [34]	10	M	Both	25 ± 4	4	‘Competed in sprint and distance races at national and/or international level’	62.2 ± 6.0	NR	DS on Treadmill; Specifics NR	Rodby motorised treadmill; Pro-Ski C2 roller skis;	NR
							59.8 ± 6.0	NR	DP on Treadmill; Specifics NR	AMIS 2001 model C metabolic system	
Berg et al. [35]	13	M	NR	24 ± 4	4	‘Participation in World Cup races or other high ranked FIS races in the 2015/2016 season’	75.5 ± 4.2	NR	Running on Treadmill; Continuous ramp. Start: Individual speed (RPE = 12) incline NR. Speed increased by 1 km.h^−1^ each minute	Forcelink motorised treadmill (RUN); Modified Concept 2 SkiErg ‘seated with ‘middle dampe setting’ (DP); Jager Oxycon pro metabolic system with mixing chamber r	‘$\dot{V}$O_2_ Peak was reported as the mean of the three highest 10 s values during the last minute of the test’^b^
							55.1 ± 6.1	NR	DP on SkiErg (UB); Continuous ramp. Start: Individual power (RPE = 12). Power increased 20 W. min^−1^		
	12	M	NA	26 ± 3	1	‘Physically active controls’	57.2 ± 6.4	NR	Running on Treadmill; As above		
							35.8 ± 4.6	NR	DP on SkiErg (UB); As above		
Bergh [22]	5	M	Dist^c^	NR^a^	5	‘Olympic and/or World Champions or medalist’	83.8 ± 6.4	6.54 ± 0.48	Running on Treadmill; Uphill on a motorised treadmill. Specifics NR	Indeterminate motorised treadmill; 100-200 l	NR
	34	M		NR^a^	4	‘Less successful international competitors’	79.6 ± 3.4	5.50 ± 0.39		Douglas bags; gas content measured by MGA200 mass spectrometer; volume measured by indeterminate spirometer	
				NR^a^	4	‘No Olympic Games or World Championship medallists, but Swedish National Champions’	71.2 ± 4.3	3.75 ± 0.25			
	9	F									
	9	F		NR^a^	3	‘Less successful skiers’	68.4 ± 2.7	3.82 ± 0.38			
Bilodeau et al. [36]	10	M	Dist^c^	21 ± 2	3	‘Members of the Pierre-Harvey National Cross-Country Skiing Centre’	77.6 ± 1.2	NR	Running on Treadmill; Continuous ramp. Start: 5.5 km.h^−1^ and 3.0° incline. Speed increased by 1.1 km.h^−1^	Indeterminate motorised treadmill; SMI Inc SkiErg; Applied Electrochemistry	NR
	10	F	Dist^c^	21 ± 2	3		62.7 ± 1.7	NR	each 2 min until 13.0 km.h^−1^ after which, incline increased by 1.8° every 2 min	open-circuit gas content analyser; Flesch Type Hewlett-Packard pneumotachometer with mixing chamber	
	10	M	Distc	21 ± 2	3		61.8 ± 4.0	NR	DP SkiErg (WB); Continuous ramp. Start: 1.4 kg load. Flywheel target cadence of 400 RPM throughout		
	10	F	Dist^c^	21 ± 2	3		47.6 ± 2.2	NR	Load increased by 0.2 kg every 2 min		
Binek et al. [37]	10	M	NR	22 ± 3	4	‘Polish national team’	65.7 ± 7.3	NR	Running on Treadmill; Incremental test. Start: 6.0 km.h^−1^ and 0.0% incline. Speed increased by 2.0 km.h^−1^ each 3 min until 14.0 km.h^−1^ after which, incline increased by 2.0% every 2 min	Indeterminate motorised treadmill; Jaeger Oxycon metabolic system	NR
	6	F		24 ± 3	4		53.8 ± 5.0	NR			
Björklund et al. [38]	6	M	NR	28 ± 4	5	‘Participated in FIS World Cup race within 12 months including 3 Olympic and World Championship medallists’	73.0 ± 3.0	5.60 ± 0.60	DS on Treadmill; Continuous ramp. Start: 11.0 km.h^−1^ (PCF 5) or 10 km.h^−1^ (PCF 3) and 4.0° incline. Incline increased by 1.0 each minute until 11.0 after which, speed increased by 0.3 km.h^−1^ every 30 s	Rodby treadmill; Pro-Ski C2 roller skis; AMIS 2001 model C metabolic system	‘Three highest values measured with a sampling duration of 10 s were averaged to calculate $\dot{V}$O_2Max_’
	6	M	NR	35 ± 4	3	‘Previous national/international experience but not participated in a FIS World Cup race within 12 months’	61.0 ± 5.0	4.90 ± 0.40			
Bolger et al [39]	4	M	NR	28 ± 3	5	FIS Points: 4 ± 4	81.7 ± 4.0	NR	Running; Specifics NR	Specifics NR	NR
	5	F	NR	26 ± 4	5	FIS Points: 8 ± 5	71.0 ± 3.9	NR			
Bucher et al. [40]	17	M	NR	22 ± 4	3	‘Ten athletes were described as elite and 29 as highly trained’^d,e^	70.1 ± 4.5	NR	DS on Treadmill; Continuous ramp. Start: M = 8.6 km.h^−1^, F = 6.8 km.h^−1^ and 9° incline. Speed increased by 0.4 km.h^−1^ every minute for the first three minutes (M = 0.5 km.h^−1^ for the first two minutes). Speed then increased every 30 s	Indeterminate motorised treadmill; Indeterminate roller-skis; 100L Douglas bag; AEI Technologies O2 (S-3A/I) and CO2 (CD-3A) analysers; Hugo Sachs Elektronik dry gas meter	‘$\dot{V}$O_2max_ was defined as the highest $\dot{V}$O_2_ measured in one bag, meeting the criteria of a filling time of > 25 s and an increase in $\dot{V}$O_2_ from the previous bag of < 150 mL min^−1^’
	10	F	NR	22 ± 4	3		58.8 ± 4.4	NR			
Bucher et al [41]	16	M	NR	19 ± 3	3	‘Required to compete at national level with 5 years minimum of ski specific training’	62.2 ± 6.9	NR	Running on Treadmill; Continuous ramp. Start: Individualised speed (specifics NR) and 10.5% incline. Speed increased by 1.0 km.h^−1^ every minute	Indeterminate motorised treadmill; Cortex Metalyzer 3b metabolic system	‘Average of the 3 highest 10 s consecutive measurements determined $\dot{V}$O_2Max_’
Carlsson et al. [42]	12	M	Dist	24 ± 4	3	FISDist Points: 122 ± 58	70.3 ± 4.2	5.34 ± 0.34	DS on Treadmill; Continuous ramp. Start: 11.0 km.h^−1^ and 4.0° incline. Incline increased by 1.0° each minute until 10.0° after which, speed increased by 0.5 km.h^−1^ every 30 s	OJK-2 motorised treadmill; Pro-Ski C2 roller skis; Jager Oxycon pro metabolic system	‘$\dot{V}$O_2Max_ was defined as the highest mean $\dot{V}$O_2_ during a period of 20 s’
Carlsson et al. [13]	8	M	Spr	25 ± 5	3	FISSpr Points: 96 ± 27	NR	5.57 ± 0.52	DS on Treadmill; Continuous ramp. Start: 11.0 km.h^−1^ and 4.0° incline. Incline increased by 1.0° each minute until 10.0° after which, speed increased by 0.5 km.h^−1^ every 30 s	OJK-2 motorised treadmill; Pro-Ski C2 roller skis; Jager Oxycon pro metabolic system	‘$\dot{V}$O_2Max_ was defined as the highest mean $\dot{V}$O_2_ during a 60 s period’
Carlsson et al. [14]	10	F	Both	25 ± 3	4	FISDist Points: 93 ± 28	63.0 ± 3.0	4.00 ± 0.30	DS on Treadmill; Continuous ramp	HP Cosmos Saturn	‘$\dot{V}$O_2Max_ was defined as the highest mean $\dot{V}$O_2_ during a 60 s period’
						FISSpr Points: 116 ± 60			Start: 10.9 km.h^−1^ and 2.4° incline. Speed increased by 0.1 km.h^−1^ and incline increased by 0.4° every 30 s	450/300rs motorised treadmill; Pro-Ski C2 roller skis; Jager Oxycon pro metabolic system	
Carlsson et al. [43]	24	M	NR	21 ± 3	4	‘Competing at the national and international levels’	71.5 ± 6.4	5.39 ± 0.57	DS on Treadmill; Continuous ramp. Start:10.0 km.h^−1^ and 3.0° incline (lower ranking skiers: n = 8)/11.0 km.h^−1^ and 4.0° incline (higher ranking skiers: n = 16). Incline increased by 1.0° each minute until 10.0° after which, speed increased by 0.5 km.h^−1^ every 60 s (lower ranking skiers)/30 s (higher ranking skiers)	OJK-2 or Rodby RL3500 motorised treadmill^c^; Pro-Ski C2 roller skis; Jager Oxycon pro metabolic system	‘$\dot{V}$O_2Max_ was defined as the highest mean $\dot{V}$O_2_ during a 60 s period’
Danielsen et al. [44]	9	F	NR	24 ± 5		‘Norwegian national- level’	NR	5.45 ± 0.64	Running on Treadmill: Specifics NR	Indeterminate motrised treadmill; Jaeger Oxycon Pro metabolic system	NR
Drzazga et al. [45]	6	M	NR	23 ± 3	4	‘Polish national team’	64.0 ± 5.9	4.68 ± 0.55	Running on Treadmill: Continuous ramp. Start: 6.0 km.h^−1^ and 0% incline. Speed increased by 2.0 km.h^−1^ every 3 min until 14.0 km.h^−1^ after which, incline increased by 2.5% every three minutes	Indeterminate motorised treadmill; Jager Oxycon metabolic system	NR
Evertsen et al. [46]	11	M	Dist^c^	NR^a^	3	‘Competing at national and international junior level’	73.0 ± 4.0	NR	Running on Treadmill; Continuous ramp. Start: Speed NR and 3.0° incline. Speed increased by 0.5 km.h^−1^ every 30 s	Woodway motorised treadmill; Jager EOS- Sprint metabolic system	‘Calculated the $\dot{V}$O_2_ as averages over 30 s intervals’
	9	F	Dist^c^		3		58.0 ± 2.0	NR			
Fabre et al. [47]	7	M	NR	21 ± 5	3^f^	‘Regional to national class’	67.4 ± 7.8	NR	Running on Treadmill; Continuous ramp. Start: 5.0 km.h^−1^ and 0.0% incline. Subsequent 1-min stages at 7.0, 9.0, 10.5, and 12.0 km.h^−1^ and 0.0% incline after which, incline increased by 2% every minute	S2500 HEF Techmachine motorised treadmill; Swenor Carbonfibre roller- skis; Sigma Sport BC800 speedometer; Cosmed K4b^2^ metabolic system	NR
							64.4 ± 8.4	NR	DS Roller-Ski (3 km asphalt road course); Start: 5.0 km.h^−1^, 8.0° incline, 1134 m altitude. Speed increased by 1.0 km.^−1^ every minute by bicycle		
Gejl et al. [48]	10	M	Both	25 ± 4	4	‘Participated in national and/or international competitions’	64.9 ± 4.1	5.10 ± 0.50	DS on Treadmill; Continuous ramp. Start: 9.0 km.h^−1^ and 7.0° incline. Speed increased by 0.5 km.h^−1^ every 45 s	Indeterminate motorised treadmill; Indeterminate roller-skis; AMIS 2001 model C metabolic system	‘Average of the three highest 10 s consecutive values was defined as $\dot{V}$O_2Max_’
							62.5 ± 4.2	5.00 ± 0.50	DP on Treadmill; Continuous ramp. Start: 21.0 km.h^−1^ and 1.0° incline. Speed increased by 1.0 km.h^−1^ every minute		
Grasaas et al. [49]	12	M	NR	25 ± 3	4	FIS Points 68 ± 27	71.4 ± 3.4	5.50 ± 0.60	V2 on Treadmill^c^; Continuous ramp. Start: 15.8 km.h^−1^ and 5.0% incline. Speed increased by 2.2 km.h^−1^ until 20.2 km.h^−1^ after which speed increased by 1.1 km.h^−1^ every minute	Bonte Technology motorised treadmill; Startex Start Skating 80 roller-skis; Jager Oxycon pro metabolic system	$\dot{V}$O_2_ measured every 10 s, the average of the three highest Consecutive values determined the $\dot{V}$O_2Peak_’^b^
Haymes and Dickinson [50]	10	M	Dist^c^	23 ± 2	4	‘United States ski team’	NR	5.34 ± 0.44	Running on Treadmill; Continuous ramp. Start: Individualised speed between 12.0–15.0 km.h^−1^ with a target HR of 160 b min^−1^. Start incline NR. Incline increased by 4% after two minutes then by 2% every two minutes	Indeterminate motorised treadmill; Meteorological balloons; Godart rapox O_2_ analyser; Capnograph CO_2_ analyser; Volumes measured by Parkinson Cowan CD4 gas meter	‘Analysed for the final three to four minutes of the run’
	10	F	Dist^c^	20 ± 4	4		NR	3.44 ± 0.36			
Hegge et al. [51]	16	M	NR	24 ± 4	4	‘Participated at national and international level’	71.5 ± 3.8	NR	V2 on Treadmill; Continuous ramp. Start: 16.0 km.h^−1^ and 5.0% incline. Speed increased by 2 km.h^−1^ until 20.0 km.h^−1^ after which speed increased by 1.0 km.h^−1^ every minute	Bonte Technology motorised treadmill; Startex Start Skating 80 roller-skis; Jager Oxycon pro metabolic system	‘Average of the three highest 10 s consecutive measurements determined $\dot{V}$O_2Peak_’^b^
Hegge et al [52]	8	M	NR	20 ± 2	3	FIS Points: 128 ± 48	73.9 ± 5.3	NR	Running on Treadmill; Continuous ramp. Start: Individualised speed (specifics NR) and 10.5% incline. Speed increased by 1.0 km.h^−1^ every minute	Indeterminant motorised treadmill; Jager Oxycon pro metabolic system with mixing chamber	‘Two highest consecutive 10 s $\dot{V}$O_2_ values were defined as $\dot{V}$O_2Peak_’^b^
	8	F	NR	23 ± 3	3	FIS Points: 131 ± 36	62.0 ± 1.5	NR			
Hegge et al [53]	10	M	NR	21 ± 4	3	FIS Points: 99 ± 21	73.4 ± 4.8	NR	Running on Treadmill; Continuous ramp. Start: Individualised speed (specifics NR) and 10.5% incline. Speed increased by 1.0 km.h^−1^ every minute	Indeterminant motorised treadmill; Jager Oxycon pro metabolic system with mixing chamber	‘Two highest consecutive 10 s $\dot{V}$O_2_ values were defined as $\dot{V}$O_2Peak_’^b^
	10	F	NR	23 ± 4	4	FIS Points: 99 ± 26	65.4 ± 4.7	NR			
Hoff et al. [54]	15	F	Dist^c^	18 ± 0	2	‘Competing at regional level’	55.3 ± 1.3	3.30 ± 0.10	Running on Treadmill; Discontinuous incremental. Start: Individualised speed at ~ 60% $\dot{V}$O_2Max_ based on previous laboratory testing, and 6° incline. 5 min stages with 20 s BLa sampling. Speed increased 1.0 km.h^−1^ every stage	Indeterminant motorised treadmill; Jager Ergo Oxyscreen metabolic system	NR
Holmberg et al. [55]	7	M	NR	27 ± 3	5	‘Included five World Championship and/or Olympic medallists’	NR	6.23 ± 0.47	DS on Treadmill; Continuous ramp. Start: 11.5 km.h^−1^ and 4° incline.Incline increased by 1° every minute	Spectra Refox motorised treadmill; AMIS 2001 metabolic system with mixing chamber; Pro-Ski C2 roller-skis (DS & DP)	‘Average of the three highest 10 s consecutive Measurements was used’
								6.00 ± 0.42	Running on Treadmill; Continuous ramp. Start: Speed NR and 4.5° incline. Speed increased by 1.0 km.h^−1^ every minute		
								5.36 ± 0.28	DP on Treadmill; Continuous ramp. Start: 15.0 km.h^−1^ and 2° incline. Incline increased by 0.5° every minute		
Ingjer [11]	6	M	Dist^c^	27 ± 3	5	‘All among best on world cup list and won World Cup races’	85.6 ± 1.6	6.38 ± 0.27	Running on Treadmill; Continuous ramp. Start: Individualised speed (specifics NR) and 10.5% incline	Indeterminant motorised treadmill; Douglas bag; Beckman E2 Oxygen analyser; Beckman IR215A Carbon Dioxide analyser;	NR
	6	F	Dist^c^	23 ± 2	5		70.1 ± 1.8	4.28 ± 0.25			
	7	M	Dist^c^	24 ± 3	4	‘Participating in international races and World Cup races’	81.5 ± 2.1	5.94 ± 0.39	Speed increased by 1.0 km.h^−1^ every minute		
	6	F	Dist^c^	23 ± 2	4		70.6 ± 3.5	3.84 ± 0.23			
	13	M	Dist^c^	23 ± 2	4	‘Occasionally participating in World Cup races, earning none or only a few World Cup points’	79.4 ± 2.9	5.74 ± 0.30			
	13	F	Dist^c^	23 ± 1	4		64.2 ± 1.9	3.84 ± 0.21			
Ingjer and Maher [56]	35	M	Dist^c^	26 ± NR	4	‘Participated in international competitions (World Cup)’	82.1 ± 2.7	6.02 ± 0.04	Running on Treadmill; Continuous ramp. Start: Individualised speed (specifics NR) and 12° incline. Speed increased by 1.0 km.h^−1^ every minute	Indeterminant motorised treadmill; Douglas bag; Beckman E2 Oxygen analyser; Beckman IR215A Carbon Dioxide analyser; Wilhelm Ritter KG wet gas meter	NR
Johansen et al [57]	14^ g^	M	NR	22 ± 2	3	‘National level’	71.4 ± 1.9	5.51 ± 0.27	Running on Treadmill; Continuous ramp. Start: F = 8.0 km.h-1, M = 10.0 km.h^−1^ and 6.0% incline. Incline raised to 7.0–10.0% within 2 min “*depending on the subjects’ fitness*”. Speed increased by 0.5 km.h^−1^ every 30 s	Rodby RL25000E motorised treadmill (DP); Woodway PPS55 motorised treadmill (RUN); Swenor wheel type 2 Fibreglass roller-skis; SensorMedics 229 metabolic system with mixing chamber	‘Defined as the mean of the highest two consecutive 20 s measurements of $\dot{V}$O_2_’
							57.9 ± 2.7	4.47 ± 0.34	DP on Treadmill; Continuous ramp. Start: Speed 2.0–4.0 km.h^−1^ below 80% of expected HRMax, determined by submaximal testing, and 4.0% incline. Incline increased by 0.5% until 80% expected HRMax reached after which, speed increased by 0.5 km.h^−1^ every 30 s		
Johansen et al. [58]	12	M	NR	24 ± 2	3	‘National level’	72.8 ± 5.5	NR	Running on Treadmill; Continuous ramp. Start: 8.0–12.0 km.h^−1^ (specifics NR) and 6.0% incline. Incline increased 1.0% every 30 s until 8.0% after which, speed increased by 0.5 km.h-1 every 30 s	Woodway PPS55 motorised treadmill (RUN); Cortex Metalyzer II metabolic system	‘Measurement every 10 s. Mean of the three highest consecutive $\dot{V}$O_2_ measurements were used’
	9	M	NR	NA^g^	3		NR	5.11 ± 0.06			
	9^h^	F	NR	^27 ± 1^	3		62.3 ± 1.1	4.34 ± 0.29			
Klusiewicz and Cempa [59]	2	M	NR	32 ± 5	4	‘Polish national team’	58.2 ± 1.6	NR	Running on Treadmill; Incremental. Start: F = 10.0 km.h-1, M = 12.0 km.h-1 and 1.5% incline. 5 min stages. Speed increased by 2.0 km.h-1 each stage until F = 16.0 km.h-1, M = 18.0 km.h-1. After which, incline increased by 1.5% each stage	Indeterminate motorised treadmill; SensorMedics Vmax29 metabolic system	NR
	4	F	NR	23 ± 2	4		55.7 ± 3.9	NR			
Kravitz et al.[60]	9	M	NA	29 ± 8	1	‘Recruited from community health clubs, faculty staff members and students’	49.9 ± 7.5	3.90 ± 0.70	Running on Treadmill; Continuous ramp. Start: Individual speed (specifics NR), “level running” (0.0% incline). Speed increased by 1.6 km.h-1 until “a predetermined self-selected jogging pace” reached after which, incline increased by 1% every minute	Quinton Q65 motorised treadmill; Jaeger Ergo- oxyscreen metabolic system	‘Recorded every 30 s’
	9	F		29 ± 8	1	Recruited from community health clubs, faculty staff members and students’	44.6 ± 3.7	2.60 ± 0.30			
Lindinger et al. [61]	12	M	NR	23 ± 2	4^i^	‘Members of the Swedish Junior and Swedish National team’	73.1 ± 4.5	NR	DS on Treadmill; Specifics NR	Rodby motorised treadmill; AMIS 2001 metabolic Indeterminate roller-skis; system	NR
Losnegard et al. [62]	6	M	NR	21 ± 3	3	‘All top 30(F)/70(M)/15(J)c in Norwegian National Championships – Experimental Group’	67.3 ± 5.1	NR	Running on Treadmill; Continuous ramp. Start: M = 10.0 km.h-1, F = 8.0 km.h-1 and 10.5% incline. Speed increased each minute (speed NR)	Rodby motorised treadmill (V1); Woodway motorised treadmill (RUN); Swenor with Type 1 Wheels roller skis; Jager Oxycon Pro metabolic system	‘Highest oxygen value averaged over 1 min was considered as $\dot{V}$O_2Max_’
	3	F	NR	21 ± 5	3		61.5 ± 1.1	NR			
	5	M	NR	21 ± 3	3	‘Control Group’	69.5 ± 4.7	NR			
	3	F	NR	23 ± 2	3		57.9 ± 2.8	NR			
	6 3	M F	NR NR	21 ± 3 21 ± 5	3 3	‘Experimental Group’	64.6 ± 5.1 56.8 ± 1.6	NR NR	V1 on Treadmill; Continuous ramp. Start: M = 10.8 km.h-1, F = 9.0 km.h-1 and 5–6° incline. Incline increased 1° every minute until 8° after which, speed increased by 0.9 km.h-1 every minute		
	5	M	NR	21 ± 3	3	‘Control Group’	68.4 ± 6.1	NR			
	3	F	NR	23 ± 2	3		53.1 ± 3.0	NR			
Losnegard et al. [63]	14	M	NR	24 ± 3	4	‘One World Cup winner, 6 skiers with top 30 results in World Cup races’	71.8 ± 3.5	5.66 ± 0.40	V1 on Treadmill; Continuous ramp. Start: 10.8 km.h-1 and 6° incline Incline increased 1° every minute until 8° after which, speed increased by 0.9 km.h-1 every minute	Rodby motorised treadmill; Swenor Skate roller-skis; Jager Oxycon pro metabolic system	‘Highest mean values over 1 min was taken as $\dot{V}$O_2Max_’
							71.5 ± 3.3	5.65 ± 0.50	V2 on Treadmill; Continuous ramp. Start: 10.8 km.h-1 and 6° incline Incline increased 1° every minute until 8° after which, speed increased by 0.9 km.h-1 every minute		
Lundgren et al [8]	17	M	NR	23 ± NR	4	‘Competing at national or international level’	77.8 ± 9.3	5.80 ± 0.31	Running on Treadmill; Continuous ramp. Start: Speed NR and 10.5% incline. Speed increased 1.0 km.h^−1^ every minute	Woodway motorised treadmill; Jager Oxycon pro metabolic system with mixing chamber	‘Mean of the three highest consecutive 10 s was used to determine $\dot{V}$O_2Max_’
Mahood et al [12]	13	M	NR	20 ± 3	3	‘All NCAA Division I athletes’	66.7 ± 4.6	NR	Various Skate Skiing Techniques in Field; ‘*most used the V2 technique* *until the final uphill section, when* *they switched to the V1 technique…’;* 3 km course, 2 km *‘relatively flat’* final kilometre at 10–15% incline. 0–1 km *‘below race pace’,* 1–2 km *‘race* *pace’,* 2–3 km *‘all-out effort to* *volitional exhaustion. None of the* *subjects completed the entire 3 km…’*	Pro-Ski inc. R2 Roadskater roller-skis; Aerosport KB1- C metabolic system	NR
Mognoni et al [64]	7	M	NR	20 ± 3	3	‘At least 4-years history of active competition at a national level’	65.9 ± 2.0	NR	Running on Treadmill with Poles (alt 1000 m); Incremental protocol. Start: M = 7.5 km.h^−^, F = 7.0 km.h^−1^ and 0° incline. Incline increased by 2° every 3 min until 4°. Speed then increased by 0.5 km.h^−1^ for 3 min after which, incline increased by 2° every 3 min	Indeterminate motorised treadmill; Medgraphics CPX/D Systems metabolic system	‘Linear regressions were computed between HR and $\dot{V}$O_2_ for each subject’
	7	F		22 ± 3	3		58.1 ± 4.2	NR			
Myakinchenko et al. [65]	27	M	NR	25 ± 5	4	‘Russian national team FIS Point Range: 7–202’	77.1 ± 4.1	NR	Running on Treadmill; Continuous ramp. Start: Speed 6.0 km.h^−1^ and 0% incline. Incline increased to 16% within 45 s after which, speed increased to 9.0 km.h^−1^ for 30 s and then further to M = 12.0 km.h^−1^, F = 11.0 km.h^−1^	Fitness Master Fitnex motorised treadmill; Cortex Metalyzer 3B metabolic system	‘Highest 30 s $\dot{V}$O_2_ value was recorded as $\dot{V}$O_2Max_’
	27	F		24 ± 3	4	‘Russian national team FIS Point Range: 6–201’	66.1 ± 3.5	NR			
Myakinchenko et al. [66]	14	M	NR	27 ± 4	4	‘Russian national team FIS Point Range: 2–45’	79.4 ± 3.5	NR	Running on Treadmill; Continuous ramp. Start: Speed 6.0 km.h^−1^ and 0% incline. Incline increased to 16% within 45 s after which, speed increased to 9.0 km.h^−1^ for 30 s and then further to M = 12.0 km.h^1^, F = 11.0 km.h^−1^	Specifics NR	NR
Mygind et al [67]	18	M	Dist^c^	24 ± NR	4	‘All Danish national team for 1987 World Cup’	66.3 ± 1.8	4.92 ± 0.13	DP SkiErg (WB); Start: 162 W Power increased by 15 W every 2 minutes until 192 W after which, participants instructed to ski ‘*flat out*’	Bespoke ‘*multipurpose’* ergometer; Jaeger Ergo Oxyscreen metabolic system	‘$\dot{V}$O_2Max_ was measured over the last 2 min’
Ng et al. [68]	43	M	Dist^c^	31 ± 4	2	‘Entrants of the Wintergreen race series’	56.6 ± 5.7	4.30 ± 0.50	Running on Treadmill; Specifics NR	Indeterminant motorised treadmill; Douglas bags; Beckman E-2 O2 analyser; Godart Capnograph CO2 analyser	NR
Nybäck et al. [69]	5	M	NR	22 ± 3	3	‘Competing at a national level’	71.5 ± 4.7	5.23 ± 0.40	DS on Treadmill; Continuous ramp. Start: 10.0–12.0 km.h-1 *“depending on the sex, age and skiing ability”* (Further specifics NR) and 3° incline. Incline increased by 1° every minute until 9° after which, speed increased by 0.4 km.h-1	Rodby motorised treadmill; Pro-ski C2 roller-skis; Cortex Metamax 3B metabolic system	‘$\dot{V}$O_2Max_ was accepted as the highest 30 s mean value’
	3	F		21 ± 1	3		58.4 ± 2.5	3.69 ± 0.47			
Østerås et al. [70]	13	F	NR	23 ± 4	4	FIS Points: 107 ± 25	64.9 ± 4.2	NR	Running on Treadmill; Continuous ramp. Start: Individualised speed (specifics NR) and 10.5% incline. Speed increased 1 km.h-1 every minute	Indeterminant motorised treadmill; Jaeger Oxycon pro metabolic system	‘Average of the three highest 10 s consecutive measurements determined $\dot{V}$O_2Max_
Rud et al. [71]	8	M	NR	24 ± 7	2^j^	‘Healthy recreational cross-country skiers’	66 ± 4.7	4.90 ± 0.40	Running on Treadmill; Continuous ramp. Specifics NR except duration was 4–6 min	Indeterminant motorised treadmill (RUN); Modified Concept 2 RowErg; Jager Oxycon pro metabolic system	NR
							60 ± 3.9	4.50 ± 0.40	DP SkiErg (WB); Continuous ramp. Specifics NR except duration was 4–6 min		
Sandbakk et al. [72]	8	M	Spr	26 ± 2	4	FIS Points: 50 ± 12	NR	5.80 ± 0.50	Running on Treadmill; Continuous ramp. Start: 9.0 km.h-1 and 10.5% incline. Speed increased by 1.1 km.h-1 every minute		
	8	F		24 ± 2	4	FIS Points: 49 ± 14	NR	3.60 ± 0.40			
	8	M		26 ± 2	4	FIS Points: 50 ± 12	62.2 ± 1.8	5.20 ± 0.40	DP on Treadmill; Continuous ramp. Start: 14.0 km.h-1 and 5% incline. Speed increased by 1.1 km.h-1 every minute	Woodway motorised treadmill (RUN); Bonte motorised treadmill (DP); Pro-ski roller-skis with standard wheels (DP); Oxycon pro metabolic system	‘Averages of the three consecutive 10 s intervals associated with the highest values designated as maximal values.’
	8	F		24 ± 2	4	FIS Points: 49 ± 14	53.6 ± 3.0	3.20 ± 0.20			
Sandbakk et al [73]	7	M	Both	24 ± 3	4	FISDist Points: 67 ± 26	70.6 ± 4.1	5.40 ± 0.55	V2 on Treadmill; Continuous ramp Start 16.0 km.h^−1^ and 5% incline. Speed increased by 1.0 km.h^−1^ every minute	Bonte motorised treadmill; Swenor roller-skis with standard wheels; Oxycon pro metabolic system	‘Average of the three highest consecutive 10 s values designated as $\dot{V}$O_2Peak_’^b^
						FISSpr Points: 80 ± 18					
Sandbakk et al. [74]	8	M	Spr	26 ± 2	4	FIS Points: 50 ± 12	69.5 ± 3.7	5.77 ± 0.49	V2 on Treadmill; Continuous ramp. Start: M = 15.8 km.h^−^1, F = 14.0 km.h^−1^ (and 5% incline. Speed increased by 2.2 km.h^−1^ every minute for the first 2 min after which, speed increased by 1.1 km.h^−1^ every minute	Bonte Technology motorised treadmill; Swenor roller-skis with standard wheels; Jager Oxycon pro metabolic system	‘Average of the three highest consecutive 10 s values was defined as $\dot{V}$O_2Max_’
	8	F		24 ± 2	4	FIS Points: 49 ± 14	60.8 ± 3.8	3.64 ± 0.43			
Sandbakk et al. [75]	12	M	Spr	25 ± 3	4	FIS Points: 44 ± 40	70.0 ± 2.7	5.74 ± 0.52	V2 on Treadmill; Continuous ramp. Start: 15.8 km.h^−1^ and 5% incline. Speed increased by 2.2 km.h^−1^ every minute for the first 2 min after which, speed increased by 1.1 km.h^−1^ every minute	Bonte Technology motorised treadmill; Swenor roller-skis with standard wheels; Jager Oxycon pro metabolic system	‘Average of the three highest consecutive 10 s values designated as $\dot{V}$O_2Peak_’^b^
Sandbakk et al. [4]	8	M	Spr	26 ± 4	5	FIS Points: 23 ± 12	70.6 ± 3.2	5.88 ± 0.40	V2 on Treadmill; Continuous ramp Start: 15.8 km.h^−1^ and 5% incline. Speed increased by 2.2 km.h^−1^ every minute for the first 2 min after which, speed increased by 1.1 km.h^−1^ every minute	Bonte Technology motorised treadmill; Swenor roller-skis with standard wheels; Jager Oxycon pro metabolic system	‘Average of the three highest 10 s consecutive measurements determined $\dot{V}$O_2Peak_’^b^
	8	M		25 ± 2	3	FIS Points: 101 ± 46	65.8 ± 3.4	5.44 ± 0.43			
Sandbakk et al [76]	12	M	NR	26 ± 6	3	FIS Points: 76 ± 21	73.0 ± 3.6	NR	Running on Treadmill; Continuous ramp. Start: Individual speed (specifics NR), and incline 10.5% Speed increased by 1.0 km.h^−1^ every minute	Indeterminate motorised treadmill; Cortex Metamax 3 metabolic system	‘Averages of three consecutive 10 s intervals with highest values determining maximal values.’
Sandbakk et al [77]	9	M	NR	18 ± 0	2	‘Junior elite athletes’	69.3 ± 5.8	NR	Running on Treadmill; Continuous ramp. Start: Individual speed (specifics NR) and 10.5% incline Speed increased 0.5–1.0 km.h^−1^ *“every* *time the participant attained *$\dot{V}$*O*_*2*_ *that* *was stable during a 30 s period”*	Rodby RL2500 E motorised treadmill; Jaeger Oxycon pro metabolic system with mixing chamber	‘Average of the three highest 10 s consecutive measurements determined $\dot{V}$O_2Max_’
Schmitt et al [78]	7	M	NR	23 ± 3	4	‘French national team’	69.3 ± 3.6	NR	Running on Treadmill; Continuous ‘incremental with ramp phase’. Start: 8.0 km.h^−1^, 2.0% incline and 1150 m altitude. Speed increased to 9.0 km.h^−1^ after 3 min. Incline increased by 2.0% every 3 min until 6% incline, after which speed increased to 10.0 km.h^−1^ until ventilatory threshold (VT). After VT, incline increased each minute (rate NR^k^) until 16%	Indeterminant motorised treadmill; MGC Diagnostics Ultima Cardio 2® metabolic system	‘Highest 30 s average value’
	5	F		23 ± 4	4		58.9 ± 2.5	NR			
Skattebo et al. [79]	18	F	NR	18 ± 3^ l^	2	‘All top 30 in Norwegian national cup. 3 participated in Junior World Championship - Experimental Group’	59.8 ± 4.4	3.64 ± 0.08	Running on Treadmill; Continuous ramp. Start: 7.0 km.h^−1^ and 10.5% incline. Speed increased by 1.0 km.h^−1^ every minute	Woodway Desmo-Evo motorised treadmill; Jaeger Oxycon pro metabolic system	‘$\dot{V}$O_2Max_ was taken as the highest average $\dot{V}$O_2_ over 60 s’
	14	F		17 ± 3^ l^	2	‘Control Group’	57.9 ± 2.6	3.47 ± 0.12			
Skattebo et al. [80]	6	M	Both	25 ± 3	5	FISDist Points: 16 ± 12	85.0 ± 3.4	6.38 ± 0.42	Running on Treadmill; Continuous ramp. Start: 10.0 km.h^−1^ and 6° incline. Speed increased 0.5 km.h^−1^ every 30 s	Rodby motorised treadmill; Swenor roller-skis; Jaeger Oxycon pro metabolic system	Highest continuous $\dot{V}$O_2_ during a 60 s period was defined as $\dot{V}$O_2Peak_’^k^
Solli et al. [81]	8	M	NR	20 ± 2	3	FIS Points: 165 ± 82	68.1 ± 5.3	5.28 ± 0.49	Running on Treadmill; Continuous ramp. Start: Individualised speed (85- 90% of predicted VO_2Max_) and incline 10.5%. Speed increased by 1.0 km.h^−1^ every minute	Rodby 2500E motorised treadmill; Jaeger Oxycon pro metabolic system	‘$\dot{V}$O_2Max_ was defined as the highest average of 2 consecutive 30 s measurements’^m^
	6	F		21 ± 3	3	FIS Points: 153 ± 44	63.1 ± 5.2	3.85 ± 0.29			
Solli et al. [82]	6	M	NR	22 ± 2	4	‘All competed at the national and international levels’	68.9 ± 2.9	5.55 ± 0.40	Running on Treadmill; Continuous ramp. Start: Individualised speed (85- 90% of predicted $\dot{V}$O_2Max_) and incline 6°. Speed increased by 1.0 km.h^−1^ every minute	Rodby 2500E motorised treadmill; Jaeger Oxycon pro metabolic system	‘$\dot{V}$O_2Max_ was defined as the highest average of 2 consecutive 30 s measurements’^m^
	6	F		20 ± 2	4		60.1 ± 3.3	3.71 ± 0.40			
Stadheim et al. [83]	10	M	NR	20 ± 3	3	‘Compete in the Norwegian National Cross-Country Skiing Cup’	69.3 ± 3.1	NR	Running on Treadmill; Continuous ramp. Start: 10.0 km.h^−1^ and 10.5° incline. Speed increased by 0.5 km.h^−1^ every 30 s	Woodway motorised treadmill (RUN); Thoraxtrainer Elite Poling Ergometer (DP); Jaeger Oxycon pro metabolic system;	‘Average of the two highest (30 s) measurements’^n^
							63.2 ± 4.7	NR	DP SkiErg (WB); Specifics NR		
Stadheim et al. [84]	13	M	NR	22 ± 3	3	‘Compete in the Norwegian National Cross-Country Skiing Cup’	72.6 ± 5.7	NR	Running on Treadmill; Continuous ramp. Start: 10.0 km.h^−1^ and 10.5° incline. Speed increased by 0.5 km.h^−1^ every 30 s	Woodway motorised treadmill (RUN); Thoraxtrainer Elite Poling Ergometer (DP); Jaeger Oxycon pro metabolic system;	‘Average of the two highest (30 s) measurements’^n^
							62.9 ± 6.8	NR	DP SkiErg (WB); Continuous ramp. Start: 15.0 km^−1^. Speed increased by 0.5 km.h^−1^ every 30 s		
Stöggl et al [85]	14	M	Spr	22 ± 5	5	‘Nine skiers with top-20 rankings in World Cup.’	NR	5.20 ± 0.50	DS on Treadmill; Specifics NR	Indeterminate motorised treadmill; Indeterminate roller-skis; AMIS 2001 metabolic system;	NR
Stöggl et al. [86]	12	M	Spr	22 ± 5	4	‘Austrian national team’	64.6 ± 6.0	4.88 ± 0.59	DS on Treadmill; Continuous ramp. Start: 11.3 km.h^−1^ and 4.0° incline. Incline increased by 0.8° every 30 s until 9.6° after which speed increased by 0.4 km.h^−1^ (Rate NR: ‘*gradually’*)	Pomer motorised treadmill; Pro-ski C2 roller-skis; Cosmed K4b^2^ metabolic system	‘Maximal men value of 10 successive breaths’
Stöggl et al [87]	16	M	Spr	26 ± 5	4	‘7 national and 9 international standard’	67.6 ± 5.4	NR^o^	DS on Treadmill; Specifics NR	Rodby motorised treadmill; Pro-ski C2 roller-skis; AMIS 2001 model C metabolic system	NR
Sunde et al. [88]	12	M	NR	19 ± 1	2	‘Well trained from Norwegian high schools and local high performance competitive skiers’	72.2 ± 5.1	5.22 ± 0.63	Running on Treadmill; Continuous ramp. Start: 8.0–12.0 km.h^−1^ (specifics NR) and 6% incline. Incline increased by 1% every 30 s until 8.0% after which, speed increased by 0.5 km.h^−1^ every 30 s	Woodway PPS 55 sport motorised treadmill; Cortex Metalyzer II metabolic system with mixing chamber	NR
	16	F	NR	18 ± 1	2		53.8 ± 4.4	3.54 ± 0.39			
Sylta et al. [89]	16	M	NR	26 ± 3	4	‘Norwegian national team 28 medal winners in junior or senior levels (specifics NR)’	79.8 ± 5.0	6.20 ± 0.50	Running on Treadmill; Continuous ramp. Start: Individualised speed (85- 90% of predicted VO2Max) and 3° incline. Speed increased by 1.0 km.h^−1^ every minute	Woodway ELG motorised treadmill; various Jaeger metabolic systems (Jaeger EOS Sprint 1989–2002 and Jaeger Oxycon pro thereafter)	NR
	13	F	NR	24 ± 4	4		70.3 ± 5.0	4.30 ± 0.30			
Sylta et al. [90]	12	M	NR	25 ± 3	4	‘Norwegian national team	80.9 ± 3.7	6.10 ± 0.46	Running on Treadmill; Specifics NR	Specifics NR	NR
	12	F	NR	24 ± 4	4	10 medal winners (sex specifics NR)’	70.9 ± 4.6	4.30 ± 0.32			
Talsnes et al. [91]	33	M	NR	19 ± 3	3	‘National level. FIS Points: 202 ± 91’	70.8 ± 4.7	5.05 ± 0.56	Running on Treadmill; Continuous ramp. Start: 9.0 km.h^−1^ and 10.5% incline. Speed increased by 1.0 km.h^−1^ every minute	Rodby RL 2500 motorised treadmill (RUN); Rodby RL 3500E motorised treadmill (V2); IDT Sports roller-skis with ‘category 2’ wheels; Jaeger Oxycon pro metabolic system	‘Average of the two highest consecutive 30 s measurements’
							68.1 ± 5.2	4.89 ± 0.66	V2 on Treadmill; Continuous ramp. Start: 14.0 km.h^−1^ and 5% incline. Speed increased by 2.0 km.h^−1^ each minute until 20.0 km.h^−1^ after which, speed increased by 1.0 km.h^−1^ every minute		
Tønnessen et al [92]	17	M	Dist	28 ± 4	5	‘Olympic medallist’	84.3 ± 5.2	6.42 ± 0.64	Running on Treadmill; Continuous ramp. Start: Individualised speed (85- 90% of predicted $\dot{V}$O_2Max_). Incline specifics NR. Speed increased by 1.0 km.h^−1^ every minute	Woodway ELG motorised treadmill; various Jaeger metabolic systems with mixing chamber (Jaeger EOS Sprint 1989–2002 and Jaeger Oxycon pro thereafter)	‘Highest average of two consecutive 30 s measurements’
	10	F	Dist	28 ± 5	5		72.6 ± 5.1	4.27 ± 0.30			
	7	M	Spr	26 ± 4	5		77.9 ± 2.9	6.27 ± 0.55			
	5	F	Spr	29 ± 8	5		68.6 ± 3.7	4.28 ± 0.41			
	8	M	Dist	26 ± 3	4	‘Olympic non-medallist’	82.0 ± 2.2	6.31 ± 0.31			
	12	F	Dist	25 ± 4	4		69.4 ± 2.7	4.16 ± 0.37			
	6	M	Spr	30 ± 4	4		78.5 ± 3.6	6.34 ± 0.49			
	8	F	Spr	25 ± 4	4		68.6 ± 4.1	4.18 ± 0.36			
Tortu et al. [93]	19	M	NR	20 ± 3	3	‘National Team athletes’	68.3 ± 6.8	NR	Running on Treadmill; Continuous ramp. Start: 8.0 km.h^−1^ and NR incline. Speed increased by 1.0 km.h^−1^ every minute	Indeterminate motorised treadmill; Cosmed K5 metabolic system	‘Most excellent 15 s $\dot{V}$O_2_ value obtained during the incremental test was considered as $\dot{V}$O_2Max_’
	17	F		19 ± 2	3		59.0 ± 5.6	NR			
Torvik et al [94]	24	M	NR	20 ± 4	3	‘Competed at the national level’	71.4 ± 3.1	NR	Running on Treadmill; Continuous ramp. Start: Speed specifics NR and 10.5% incline. Speed increased by 1.0 km.h^−1^ every minute	Rodby RL 2500E motorised treadmill; Jaeger Oxycon pro metabolic system	‘Mean of the three highest consecutive 10 s measurements of $\dot{V}$O_2_ at each stage was designated as $\dot{V}$O_2peak_’^b^
Torvik et al. [95]	8	M	NR	22 ± 1	3	FIS Points: 120 ± 44	69.4 ± 5.1	NR	DS on Treadmill; Continuous ramp. Start: 10.0 km.h^−1^ and 7.0% incline Incline increased by 1.0% every minute	Rodby 3500ML motorised treadmill; Swenor Fiberglass roller-skis with ‘standard resistance wheel 2’; Jaeger Oxycon pro metabolic system with mixing chamber	‘Measured every 10 s and every minute of the test, while $\dot{V}$O_2_ was calculated by the average three values closest (last 30 s) to every step change.’
							65.3 ± 6.7	NR	DP on Treadmill; Continuous ramp Start: 10.0 km.h^−1^ and 7.0% incline Incline increased by 1.0% every minute		
Undebakke et al. [96]	12	M	NR	26 ± 4	4	FIS Points: 66 ± 7	74.5 ± 4.1	5.80 ± 0.40	Running on Treadmill; Continuous ramp. Start: Individualised speed (RPE 12) based on submaximal stages, and 10.5% incline. Speed increased by 1.0 km.h^−1^ every minute	Forcelink BV motorised treadmill (DS and RUN); Modified Concept 2 SkiErg ‘seated with ‘damper setting 5’ (DP); IDT Sports classic roller- skis with category 2 wheels; Jager Oxycon pro metabolic system	‘Average of the three highest consecutive 10 s recordings during the last minute determined $\dot{V}$O_2Peak_’^b^
							75.9 ± 5.3	5.87 ± 0.40	DS on Treadmill; Continuous ramp Start: Individualised speed (RPE 12) based on submaximal stages, and 12.0% incline. Speed increased by 1.0 km.h^−1^ every minute		
							54.8 ± 6.2	4.24 ± 0.40	DP SkiErg (UB); Continuous ramp Start: Individualised power (RPE 12) based on submaximal stages. Power increased by 20 W min^−1^		
Vergès et al. [97]	10	M	NR	19 ± 2	3	‘National level’	73.7 ± 4.2	5.23 ± 0.49	Running on Treadmill; ^b^Continuous incremental. Start: 9.0 km.h^−1^ and 0.0% incline. Incline increased by 2.0% every 3 min, except each 4th step in which speed increased by 0.7–1.1 km.h^−1^	Indeterminant motorised treadmill; Brainware metabolic system	NR
Vergès et al [98]	7	M	NR	18 ± 1	3	‘National level’	NR	4.05 ± 0.90	Running on Treadmill; Continuous incremental; Speed and incline adjusted to 70%, 80%, 90% HRMax (based on previous testing) each 6 minutes. Thereafter, incline increased by 2% every 2 min	Indeterminant motorised treadmill (Running); Marwe Skating 610 roller- skis with high resistance; Brainware metabolic system (Running); Jaeger Oxycon Mobile (Roller- skiing)	‘$\dot{V}$O_2_ was obtained by averaging the last minute before exhaustion’
							NR	3.98 ± 0.75	Roller-skiing on tarred road with varied techniques; Discontinuous 4 km loops at 70%, 80%, 90% HRMax (based on previous testing) and ‘maximum speed’, separated by 10 minutes		
Walther et al.^*p*^ [99]	10	M	Both	NR^a^	5	"Classified as world-class (Tier 5) according to performance caliber classification by McKay et al.…" FISComb: 6 ± 9	81.6 ± 3.8	6.02 ± 0.50	Running on Treadmill; Continuous ramp. Start: Individualised speed (specifics NR) and 10.5% incline Speed increased by 1.0 km.h^−1^ every minute	Indeterminant motorised treadmill; Inditerminant metabolic system	NR
	7	F	Both	NR^a^	5		71.3 ± 4.4	4.46 ± 0.30			
Wang et al. [100]	12	M	NR	21 ± 2	3	‘2 national players [sic] and 4 reserve national players’	77.9 ± 1.2	NR	Running on Treadmill; Start: 2.7 km.h^−1^ and 10% incline. Speed increased by 1.3–1.5 km.h^−1^ and incline by 2% every two minutes	Indeterminant motorised treadmill; COSMED; Quark CPET metabolic system	NR
Wisløff et al. [101]	8	F	NR	19 ± 1	2	‘Competitive cross-country skiers… recruited from a secondary school’	NR	3.60 ± 0.40	Running on Treadmill; Start 9.0 km.h^−1^ and 3° incline. Speed increased by 1.0 km.h^−1^ every minute until ‘*close to* *exhaustion’* followed by supramaximal intensity stage ~ three minutes	Challenger LE5000 motorised treadmill; Jaeger EOS Sprint metabolic system	NR
Zhu et al. [102]	5	M	NR		3	‘Competitive at national level’	63.3 ± 6.5	NR	Running on Treadmill; Continuous ramp. Start: Individualised speed (specifics NR) and 10.5% incline Speed increased by 1.0 km.h^−1^ every minute	Rodby RL 1700 motorised treadmill; Indeterminate metabolic system	‘Three highest 10 s successive results were averaged to obtain $\dot{V}$O_2max_’

Both, Known to be competing in both sprint and distance disciplines; *Dist*, Distance competing athlete; *DP*, Double Poling; *DS*, Diagonal Stride; *F*, Female; *FIS*, International Skiing Federation; *FISComb*, Known to be a combined measure of sprint and distance point scores; *FISDist* Distance specific point score; *FISSpr*, Sprint specific point score; *M*, Male; *n* = , Number of participants; *NCAA*, National Collegiate Athletic Association; *NR*, Not Recorded; *PCF*, Participant Classification Framework tier; *RPE*, Rating of Perceived Exertion; *Spr*, Sprint athlete; *UB,* Upper Body; $\dot{V}$O_2Max_, Maximal Oxygen Uptake; $\dot{V}$O_2Peak_, Maximal Oxygen Uptake for Modality - see footnotes; *WB*, Whole Body

^a^Value not reported, but contextual indication that all participants were above the exclusion criterion age, i.e. > 16 years old; ^b^Refers to assessment of $\dot{V}$O_2_ plateau, therefore considered a mode-specific $\dot{V}$O_2Max_; ^c^Based on the date preceding sprint events; ^d^Numbers of each performance level not separated by sex; ^e^Unclear why so many participants data were excluded, 39 recruited but only 27 reported and 3 exclusion explanations provided; ^f^National skiing federation points reported for each individual; ^g^Data could only be separated for certain individuals, specific individual ages were not known to report here but all participants > 16 years old; ^h^Study recruited two females who did not complete the protocol, leaving exclusively male data; ^i^Ratio of junior and senior members unknown, but assumed majority senior due to reported mean age and range (20–26 years), therefore considered representative of PCF tier 4; ^j^Described as recreational (‘PCF 1’) but also stated to identify as a cross-country skier (‘PCF 2’), therefore PCF 2 applied; ^k^Rate of 2% min^−1^ assumed as a continuation of the previously described stated for the running protocol procedures; ^o^Data were reported as 5.20 ± 0.50 but due to the same participant descriptors as Stöggl et al. [85], this was considered a duplicate sample and not considered in analyses. Study included here for relative $\dot{V}$O_2Max_ data; ^p^Senior season data included; ^*p*^ > 16 years old

### Descriptive Study Details

Data for 1392 participants (Male (M): n = 990, Approximate Mean Age: 24 years; Female (F): 402, Approximate Mean Age: 22 years) were extracted. A total of 1667 data samples (Male (M): n = 1229, 73.7%, Female (F): n = 438, 26.3%) were recorded across all of the included studies. Studies were separated by exercise mode or skiing technique to determine $\dot{V}$O_2Max_ resulting in 52 studies for running (Total: n = 1040; M: n = 684, 65.8%; F: n = 356, 34.2%), 18 studies for Diagonal Stride (DS) on a treadmill (Total: n = 234; M: n = 201, 85.9%; F: n = 33, 14.1%), 1 study for DS in the field (Total: n = 7; M: n = 7, 100.0%), 8 studies for Double Poling (DP) on a treadmill (Total: n = 86; M: n = 68, 79.1%; F: n = 18, 20.9%), 6 studies for DP on a Ski Ergometer (Unrestricted) (Total: n = 84; M: n = 69, 82.1%; F: n = 15, 17.9%), 2 studies for DP on a Ski Ergometer (Upper Body Only) (Total: n = 37; M: n = 37, 100%), 2 studies for V1 (skate skiing technique [103]) on a treadmill (Total: n = 33; M: n = 25, 75.8%; F: n = 8, 24.2%), 8 studies for V2 (skate skiing technique [103]) on a treadmill (Total: n = 126; M: n = 118, 93.7%; F: n = 8, 6.3%), and 2 studies using a variety of skiing techniques in the same trial (Total: n = 20; M: n = 20, 100%). Eight studies [36, 56–58, 67, 79, 94, 100] reported multiple assessment points using the same technique, as part of their research design (longitudinal and/or cross-over intervention design), requiring data to be combined [27].

### Relative $\dot{V}$O_2Max_: Treadmill Running

**Fig. 3:**
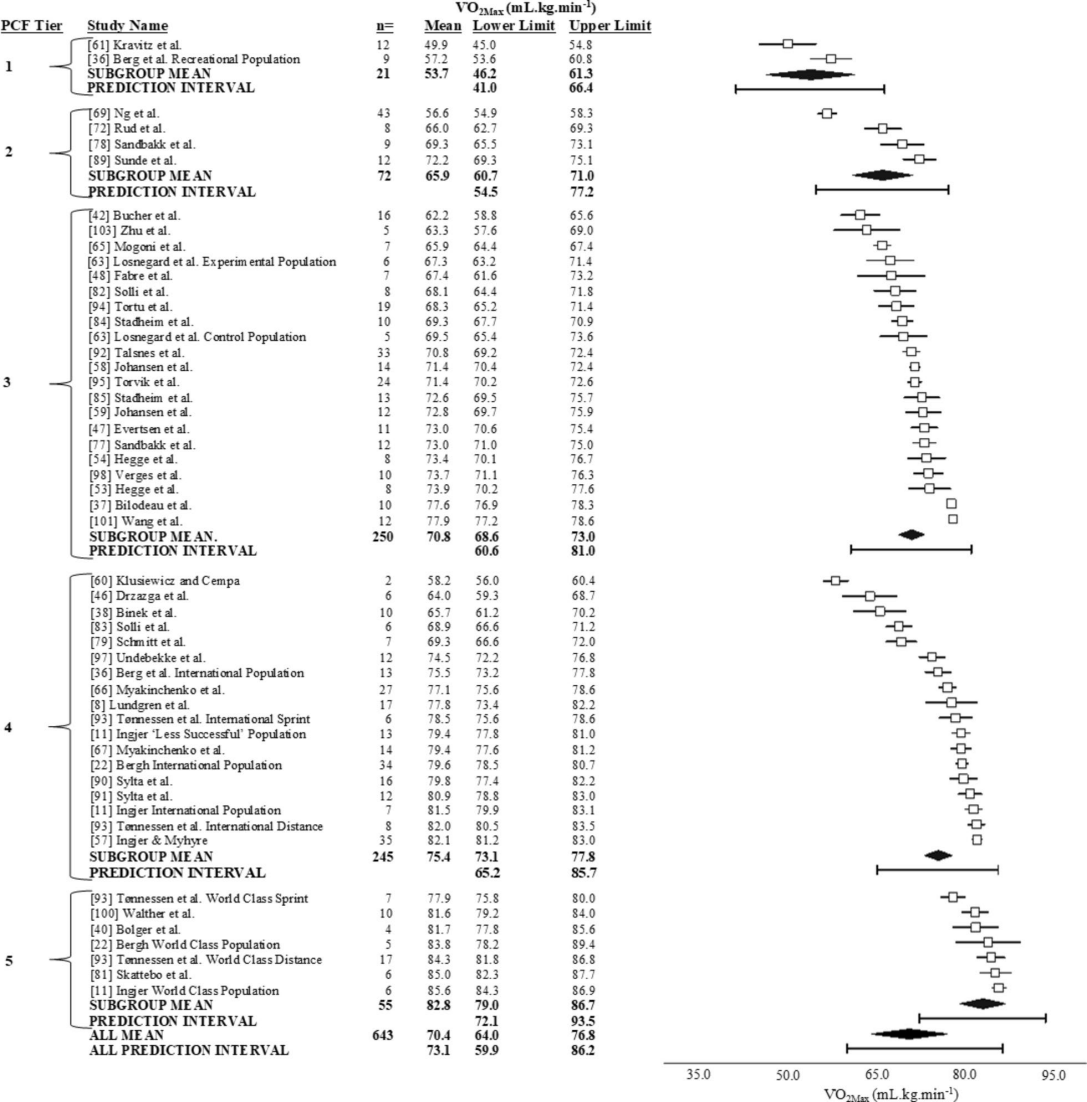
Male relative maximal oxygen uptake data for included studies, sub-grouped by participant classification framework. PCF, Participant Classification Framework; $\dot{V}$O_2Max_, Maximal Oxygen Uptake

**Fig. 4:**
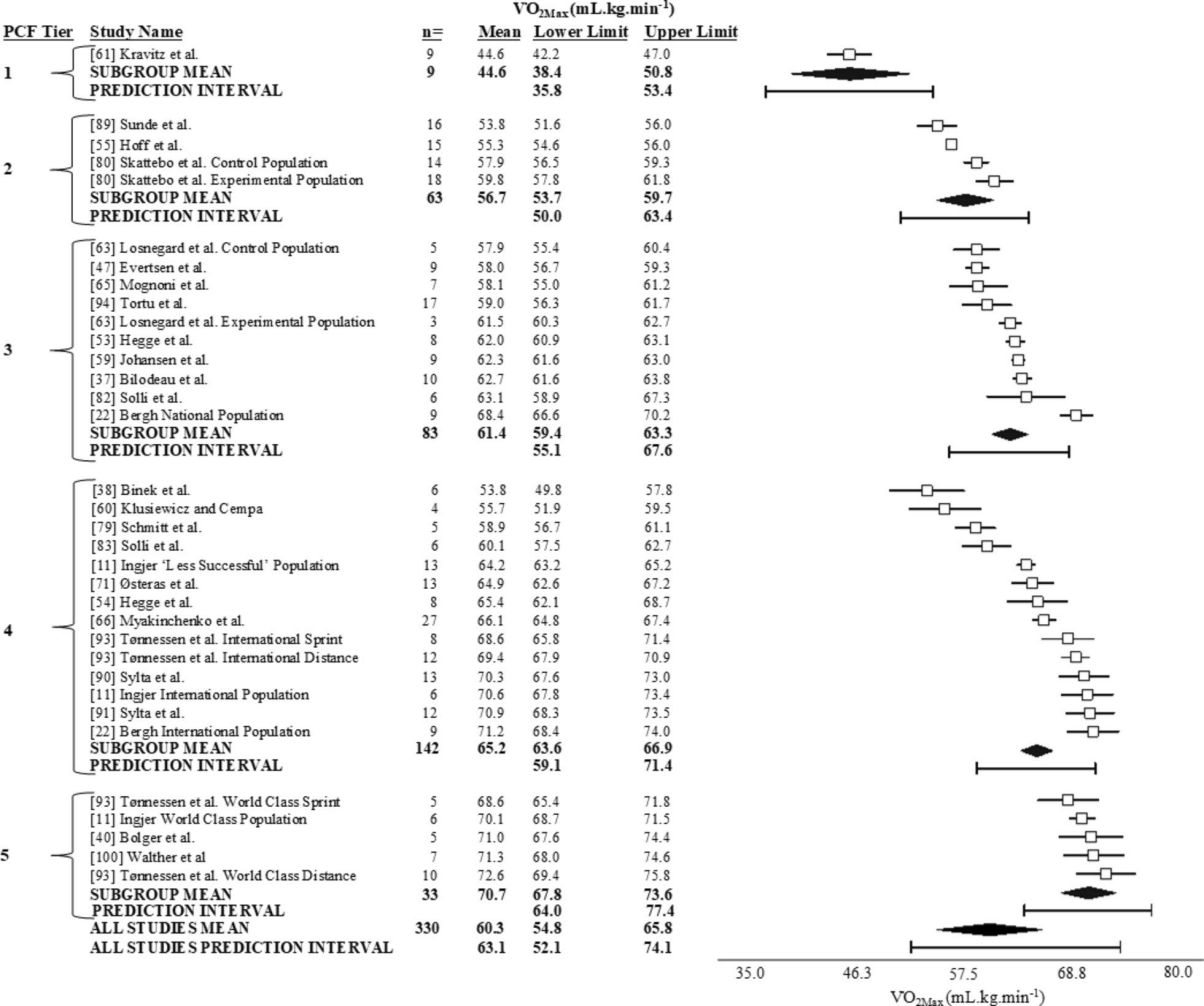
Female relative maximal oxygen uptake data for included studies, sub-grouped by participant classification framework. PCF, Participant Classification Framework; $\dot{V}$O_2Max_, Maximal Oxygen Uptake

Of the 47 studies measuring relative $\dot{V}$O_2Max_ values from treadmill running protocol in male participants, 52 groups of data were extracted and separated by PCF score. PCF explained 42% of variance in $\dot{V}$O_2Max_ (τ = 6.51, R^2^ = 0.42) and group means increased with PCF (Fig. 3; τ^2^ = 42.4). Of the 26 studies measuring relative $\dot{V}$O_2Max_ in females using a treadmill running protocol, 34 groups of data were extracted and separated by PCF score. PCF explained 70% of variance in $\dot{V}$O_2Max_ (τ = 5.36, R^2^ = 0.80) and group means increased with PCF (Fig. 4; τ^2^ = 28.7).

### Absolute $\dot{V}$O_2Max_: Treadmill Running

**Fig. 5:**
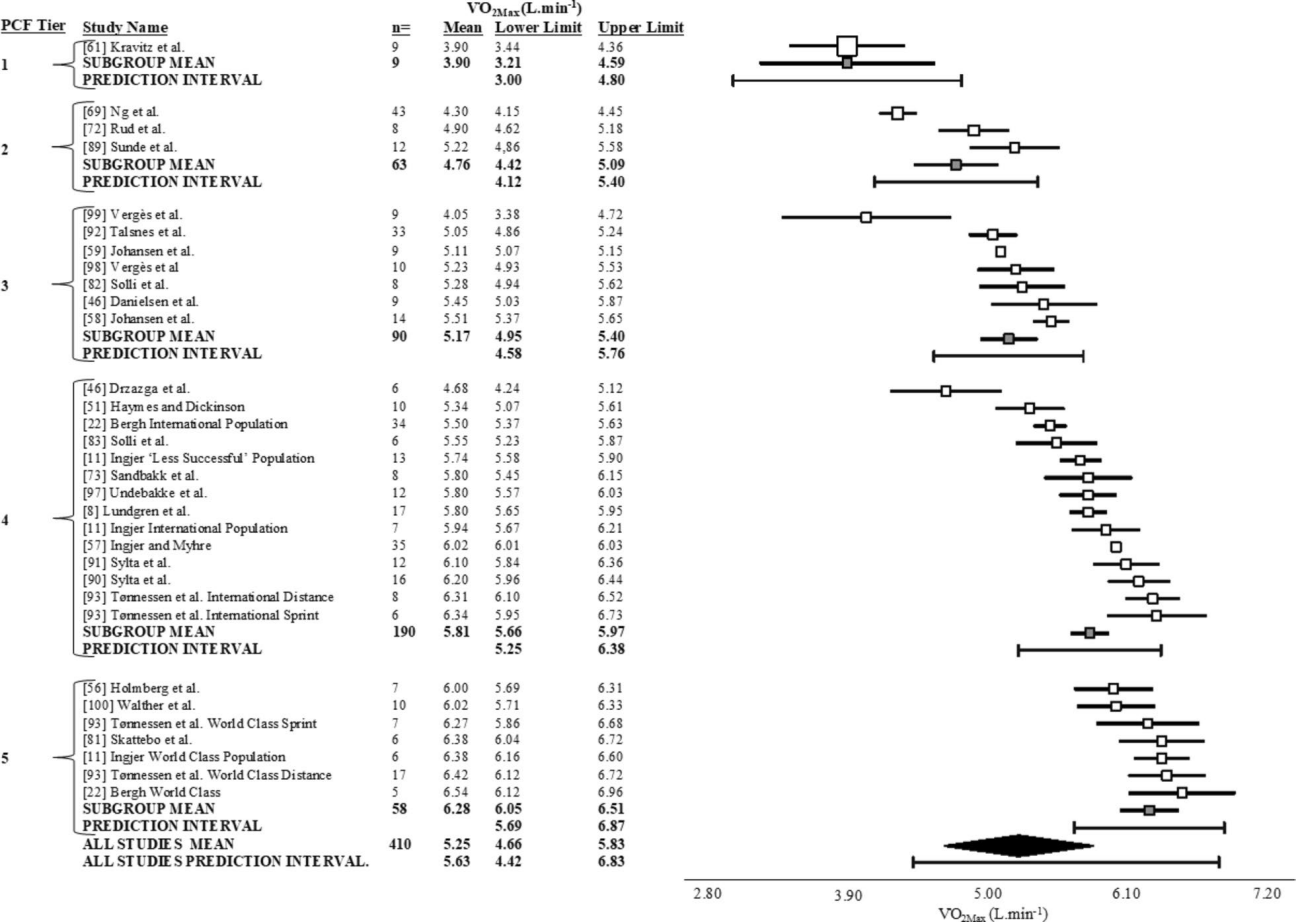
Male absolute maximal oxygen uptake data for included studies, sub-grouped by participant classification framework. PCF, Participant Classification Framework; $\dot{V}$O_2Max_, Maximal Oxygen Uptake

**Fig. 6:**
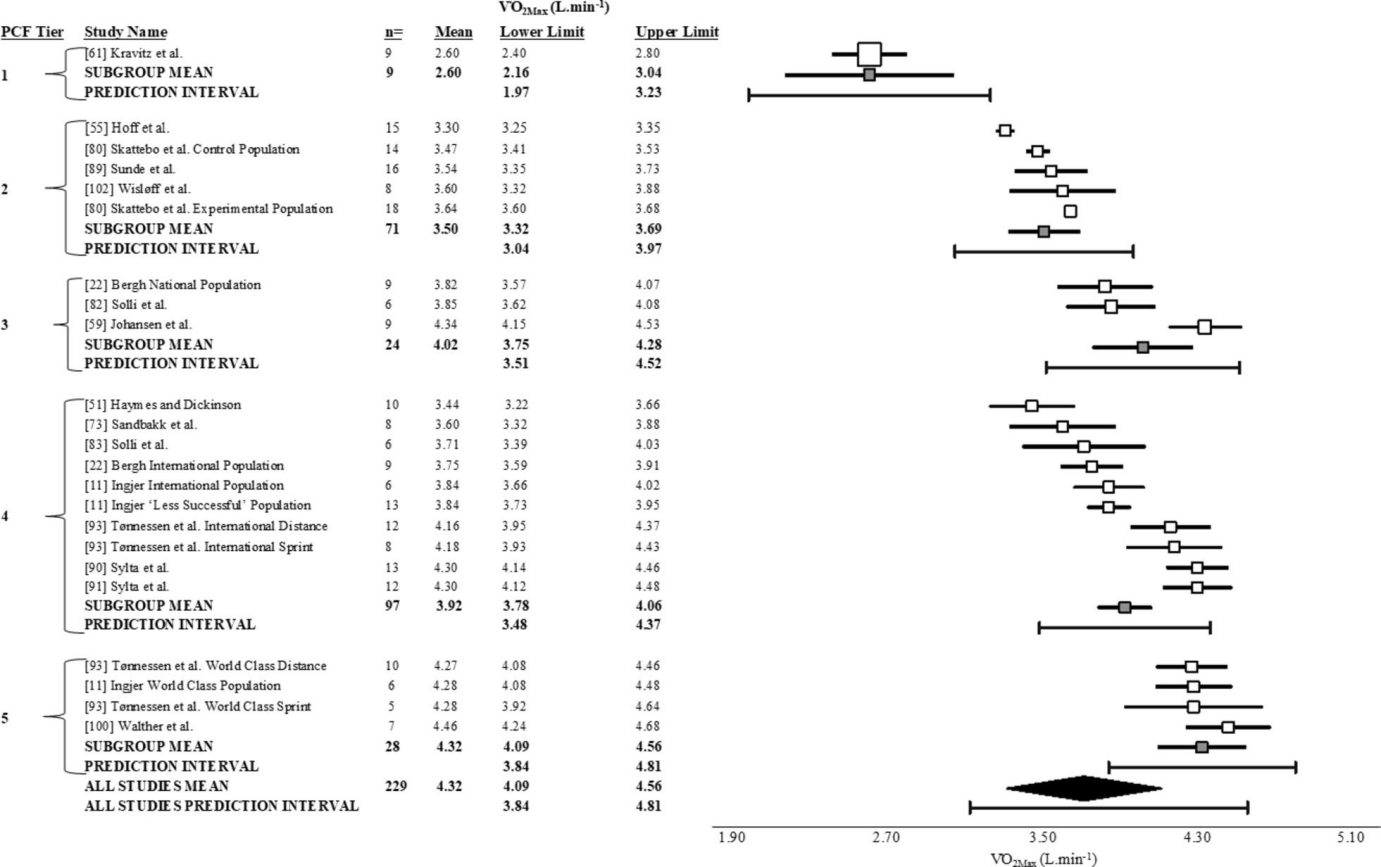
Female absolute maximal oxygen uptake data for included studies, sub-grouped by participant classification framework. PCF, Participant Classification Framework; $\dot{V}$O_2Max_, Maximal Oxygen Uptake

Of the 26 included studies measuring absolute $\dot{V}$O_2Max_ value in males using a treadmill running protocol, 32 groups of data were extracted and separated by PCF score. PCF explained 80% of variance in $\dot{V}$O_2Max_ (τ = 0.59, R^2^ = 0.80) and group means increased with PCF (Fig. 5; τ^2^ = 0.35). Of the 16 included studies measuring absolute $\dot{V}$O_2Max_ in females using a treadmill running protocol, 23 groups of data were extracted and separated by PCF score. PCF explained 66% of variance in $\dot{V}$O_2Max_ (τ = 0.34, R^2^ = 0.66) and group means increased with PCF, with the exception of tier 4. (Fig. 6; τ^2^ = 0.12).

### Benchmark $\dot{V}$O_2Max_ Values

**Fig. 7:**
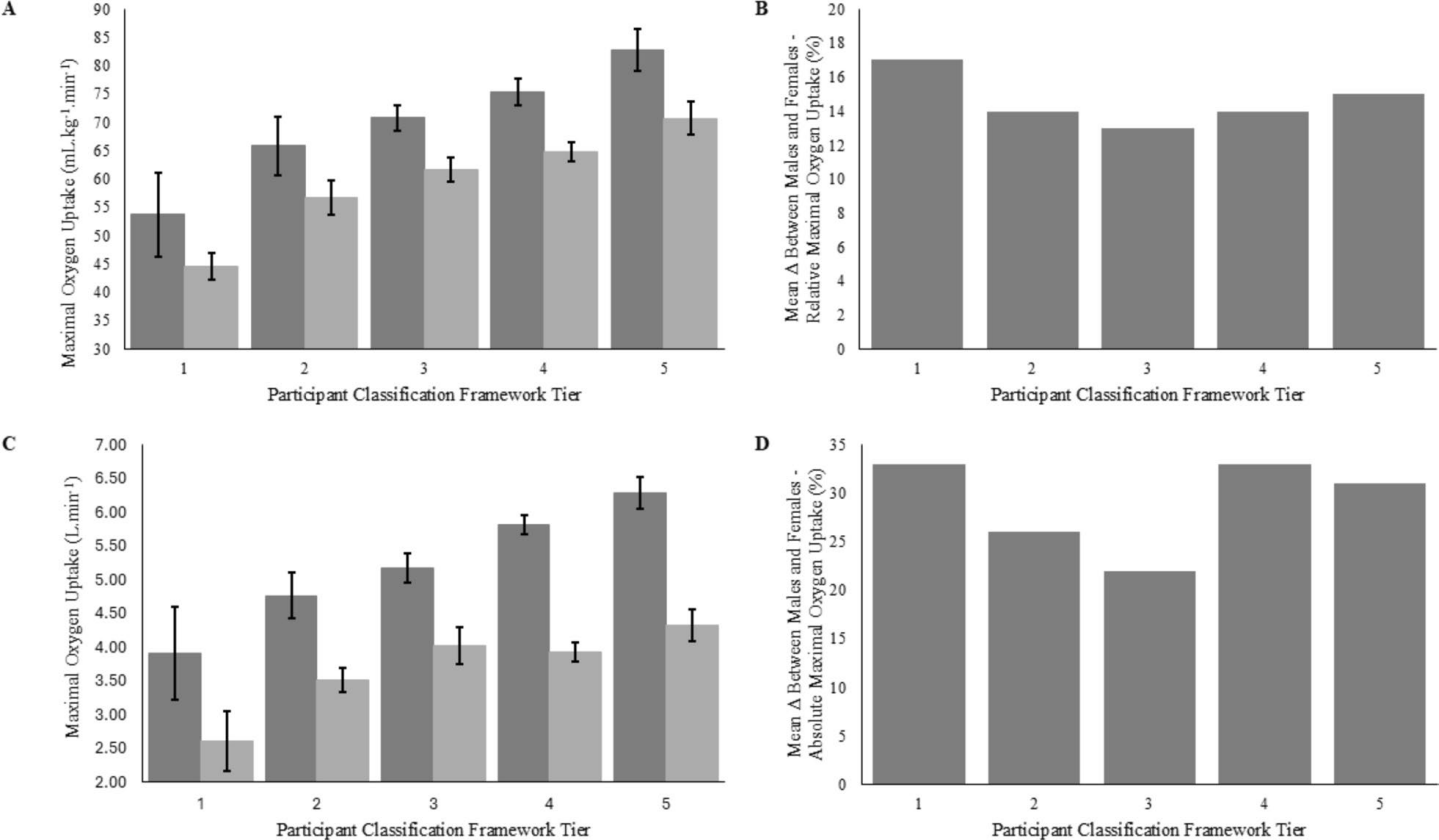
Summary statistics for participant classification framework subgroup mean averages. **A:** Maximal oxygen uptake relative to body mass. **B:** Percentage differences of mean maximal oxygen uptake relative to body mass between males and females. **C:** Absolute maximal oxygen uptake. **D:** Percentage differences of absolute maximal oxygen uptake between males and females. **•** = Males **•** = Females

Pooled group analysis of each PCF tier produced key values that may be considered benchmark $\dot{V}$O_2Max_ values and are summarised in Table 4 and Fig. 7.

**Table 4:** Key data for relative and absolute $\dot{V}$O_2Max_ benchmarking using running-based incremental exercise tests separated by Participant Classification Framework tier and sex

PCF Tier	Pooled Mean	Min	Max	SE ( ±)	95% CI		Pooled Mean	Min	Max	SE ( ±)	95% CI
*Male Relative (mL·kg*^*−1*^*·min*^*−1*^*)*						*Female Relative (mL kg-1 min-1)*					
World Class (5)	82.8	77.9	85.6	3.7	79.1, 93.5		70.7	68.6	72.6	1.5	67.8, 73.6
Elite (4)	75.4	58.2	82.1	1.2	73.1, 77.8		65.2	53.8	71.2	0.8	63.6, 66.9
National (3)	70.8	62.2	77.9	1.1	68.6, 73.0		61.4	57.9	68.4	1.0	59.4, 63.3
Development (2)	65.9	56.6	72.2	2.6	60.7, 71.0		56.7	53.8	59.8	1.5	53.7, 59.7
Recreational (1)	53.7	49.9	57.2	3.8	46.2, 61.2		44.6	44.6	44.6	4.5	35.8, 53.4
*Male Absolute (mL min*^*−1*^*)*						*Female Relative (mL min*^*−1*^*)*					
World Class (5)	6.33	6.02	6.54	0.12	6.09, 6.57		4.32	4.27	4.46	0.12	4.09, 4.56
Elite (4)	5.87	5.34	6.34	0.08	5.35, 6.40		3.92	3.44	4.30	0.07	3.78, 4.06
National (3)	5.18	4.05	5.51	0.11	4.97, 5.39		4.02	3.82	4.34	0.13	3.75, 4.28
Development (2)	4.75	4.30	5.22	0.16	4.44, 5.07		3.50	3.30	3.64	0.10	3.32, 3.69
Recreational (1)	3.90	3.90	3.90	0.34	3.24, 4.56		2.60	2.60	2.60	0.10	2.16, 3.04

*CI*, Confidence Interval; *Max*., Maximum; *Min*., Minimum; *PCF*, Participant Classification Framework; *SE*, Standard Error; $\dot{V}$O_2Max_, Maximal Oxygen Uptake

## Discussion

### Overview

This review is the first to systematically group data with retrospective use of a PCF, to quantify relationships between $\dot{V}$O_2Max_ and XCS using meta-regressions. Results established both absolute and relative $\dot{V}$O_2Max_ running measures, tend to increase with each PCF tier in males and females. The tier-wise trend was most evident in relative measurements. These findings provide a quantitative description of $\dot{V}$O_2Max_ across performance strata, and contribute empirical context to the prominent emphasis that $\dot{V}$O_2Max_ has traditionally received within XCS.

### Athlete Classification

Performance classification was a central challenge to interpreting $\dot{V}$O_2Max_ values across the performance spectrum. Studies varied considerably in their descriptions of participants and athletes (e.g. national-team status, level of competition, or general “elite” labels). Attempting to standardise athlete performance definitions, this study retrospectively applied an amended PCF (Table 1). The original framework provides broad definitions but with flexibility to enhance applicability [23]. This flexibility is not necessarily a weaknesses of the PCF, but rather demonstrates the complexity of the challenge it attempts to address.

Thresholds of FIS points based on 20th and 300th position, were calculated to assign PCF tiers 3–5 to studies reporting this value. Owing to FIS point reporting inconsistencies, ‘overall performance’ was evaluated by averaging sprint and distance points (Table 2). These values offer guidance for prospective studies using the PCF, preferably alongside confirmatory quantitative data [104]. Introduced in 1994, FIS points were initially a single score reflecting distance performance. Separate sprint points were introduced in 2000, with the majority of athletes competing in both disciplines [3]. Therefore, studies since 2000, reporting single FIS point values, may represent sprint, distance or combined values similar to the approach taken in this study. Despite these options, it often remains unclear which discipline is reported [39, 49, 52, 53].

Where quantitative competitive information was absent, classification relied on the contextual qualitative details [33, 43, 47]. Although classification in this review followed a structured, independent and blinded decision process, residual uncertainty could not be entirely eliminated. These issues underscore that classification represents a reporting limitation of the available literature rather than an evaluative judgement on individual studies, many of which were not designed with performance-tier comparison as a primary purpose.

It is also important to recognise variations in international standards across different countries. Klusiewicz and Cempa [59], and Drzazga et al. [45], both described their participants as ‘members of the Polish National Team’. A qualifying quantitative descriptor might have suggested these studies be considered more representative of national level athletes. Yet, in the absence of a quantitative description, the description of ‘National Team’ status was inferred as an international standard of performance, although seemingly far below the international average standard.

This variation of national standards, teamed with a comparative lack of female data, likely explains the observed slight drop in pooled average absolute $\dot{V}$O_2Max_ for tier 4 females compared with tier 3. The tier 3 average was calculated from just three studies [22, 58, 81]. One group of female participants in Bergh’s [22] study were scored as tier 4. In this case, it was inferred that despite not winning international medals, they had likely competed, still representing a tier 4 standard. The described ‘less successful’ athletes were then considered representative of tier 3, using the prior group as a reference standard, although these participants may have equally been representative of tier 4, also. The participants of the study by Johansen et al. [58] were more unambiguously described as being ‘national level’, whilst Solli et al. [81] reported FIS points aligning with tier 3 based on the thresholds put forward by this study. Of the 24 participants, 9 were represented within the Johansen et al. [58] study, containing Norwegian athletes, who likely represent the extreme highs of each performance tier, based on the nation’s historical success in XCS.

In more contemporary studies reporting pre-assigned PCF tiers, these too should not be regarded as self-evident. Researchers should avoid split tier reporting (i.e. between PCF Tier 2/3) and identify a single, most representative tier. For example, Holsbrekken et al. [105], reported their participants as:

“…highly trained and elite XC skiers that regularly participated in national and international races, while the recreational XC skiers should be classified as trained and highly trained.”

It is unclear which PCF tier is the most appropriate, as the language implies the competitive group included tier 3 and tier 4 athletes, without quantifying the numbers of each. Likewise, the ‘recreational’ group (itself a tier 1 descriptor) could represent tier 2 *or* tier 3 participants, with use of the phrases ‘trained’ and ‘highly trained’.

Another example of ambiguous PCF reporting is provided within Staunton et al. [106]. Their study compared a group of cyclists with a group of cross-country skiers, stating:

“These participants represent tier 4 and tier 5 level athletes according to the sports participant classification framework.”

Similar to Holsbrekken et al. [105], no details were provided about the distribution of athletes across tiers or sports. Such ambiguity undermines the PCF’s purpose of standardised classification. For this reason, these studies were excluded from this study. Future studies should prioritise clarity to maintain the framework’s usefulness.

### Defining and Calculating $\dot{V}$O_2Max_

Defining $\dot{V}$O_2Max_ and $\dot{V}$O_2Peak_ remains complex due to varied criteria, calculation methods and interpretations. $\dot{V}$O_2Max_ is often considered the limit of aerobic capacity, characterised by a $\dot{V}$O_2_ plateau despite increasing workload [16, 106, 107]. This is typically supported by secondary criteria; respiratory quotient > 1.10, blood lactate > 8.0 mmol·L, heart rate (HR) near HR_Max_, and maximal or near-maximal rating of perceived exertion [108, 109], though the widespread accuracy of these are debated [110–112]. A $\dot{V}$O_2_ plateau was the accepted definition for the purpose of this study [16]. However, it must be acknowledged that the presence of $\dot{V}$O_2_ plateau during incremental testing, is not guaranteed nor universally identifiable [113]. Consequently, $\dot{V}$O_2Peak_, the highest value recorded during testing, is often reported. Although this *may* equal $\dot{V}$O_2Max_, $\dot{V}$O_2Peak_ suggests a limit either could not be reached or could not be confirmed [110, 114]. To address this, supramaximal verification bouts have been proposed to confirm the presence of a plateau [110, 115]. However, universal use of verification bouts, and the abandonment of $\dot{V}$O_2Peak_, is also controversial [114–121].

An alternative rationale for using $\dot{V}$O_2Peak_, common in XCS is measurement variances across exercise modes and the total recruited muscle mass [1]. Studies measuring $\dot{V}$O_2_ using DP report lower ‘maximal’ values [34, 55, 72]. For example, when comparing tier 5 athletes, DP $\dot{V}$O_2_ values (5.36 L·min^−1^) were lower than those reported during DS (6.23 L·min^−1^) and running (6.00 L·min^−1^) modalities [55]. This phenomenon is even clearer in lesser trained individuals [57]. However, this convention can render such $\dot{V}$O_2Peak_ designation indistinguishable from the former justification of $\dot{V}$O_2Peak_, representing an unconfirmed $\dot{V}$O_2Max_. For this study, studies reporting $\dot{V}$O_2Peak_ with reference to a plateau were considered representative of a ‘mode specific’ $\dot{V}$O_2Max_ and therefore relevant for inclusion [4, 36, 50, 52–54, 74, 76, 81, 95, 97]. In contrast, if *only* secondary criteria were reported, these were not considered sufficient to overrule the original study definition and were excluded [6, 122–140]. It is recommended that $\dot{V}$O_2Max_ be used in cases where a $\dot{V}$O_2_ limit has been verified [109, 110], whilst $\dot{V}$O_2Peak_ remains relevant if a limit is unverified [114, 116, 117]. To account for the important consideration of muscle mass, reporting mode specific $\dot{V}$O_2Max_ (i.e. $\dot{V}$O_2MaxDP_, $\dot{V}$O_2MaxSkiErg_, $\dot{V}$O_2MaxV2,_ etc.) may reduce ambiguities when comparing across exercise modes or skiing techniques [114, 115].

In addition to challenges associated with defining $\dot{V}$O_2Max_ and $\dot{V}$O_2Peak_, calculation differences and reporting oversights may induce discrepancies when comparing values [104, 141]. Reducing the number of breaths or time used to calculate averages, increases calculated $\dot{V}$O_2Max_ by as much as 10% in untrained individuals [141]. To account for this, Martin-Rincon et al. [141] suggested conversion equations, to standardise values from different calculation approaches. However, these equations require agreed reporting standards, currently not sufficiently present for the analyses carried out in this study. Furthermore, these equations have not been verified for use within populations with exceptionally high $\dot{V}$O_2Max_ (i.e. tier 5 XCS athletes with substantially > 60 mL·kg^−1^·min^−1^). Nevertheless, such variances should be considered and may explain some variability observed in certain tiers.

### $\dot{V}$O_2Max_ in XCS

The worth of enhanced $\dot{V}$O_2Max_ for endurance performance is not a subject of debate. Clearly, athletes wishing to compete at the highest sporting level must demonstrate elevated values compared to an ‘average’ individual. Predictably then, the findings of this study demonstrate $\dot{V}$O_2Max_ increases with PCF tier in male and female cross-country skiers across heterogeneous performance standards. Interestingly, the findings of this study suggest $\dot{V}$O_2Max_ continues to distinguish ‘successful’ (Tier 5) from ‘less successful’ (Tier 4) internationally competitive XCS athletes [11, 22], which is not always the case in other endurance sports [16, 18]. Such an observation potentially highlights why $\dot{V}$O_2Max_ is so highly regarded within XCS.

Mahood et al. [12] found large correlations between $\dot{V}$O_2Max_ and ranking (r = 0.66, *p* < 0.05) and very large correlations between $\dot{V}$O_2Max_ and simulated 10 km time-trial performance (r = 0.74, *p* < 0.05). Additionally, Carlsson et al. reported very large correlations between $\dot{V}$O_2Max_ and sprint performance, measured by race speed in males (r = 0.86, *p* = 0.0069) [13] and FIS points in females (r = 0.81, *p* = 0.0043) [14]. These observations align with established exercise physiology principles: enhanced oxidative phosphorylation increases $\dot{V}$O_2_, delaying increasing anaerobic contributions and consequently fatigue, thereby improving performance [7, 16]. However, examining individual data reported in the data published by Carlsson et al. [14], suggests this study was not as homogenous as the term ‘elite’ may lead some to infer. Digitisation of these data and the thresholds set in this study, suggest one participant would have qualified as PCF Tier 5 (< 31 FIS points), four participants PCF tier 4 (< 111 FIS points), and five as PCF tier 3 (> 111 FIS points). Therefore, the finding that VO_2Max_ correlated with performance is unsurprising, demonstrating the limitation of drawing conclusions on the basis of single studies.

The $\dot{V}$O_2Max_ values of tiers 4 and 5 encompass the full spectrum of international XCS competition standards. A systematic review investigating the benchmarking of female cycling performance suggested even broader distribution of $\dot{V}$O_2Max_ in their highest two performance levels [142]. In a process not dissimilar to this study, performance level 4 individuals (n = 13) averaged 52.6 ± 3.8 mL·kg⁻^1^·min⁻^1^ and 3.10 ± 0.30 L·min⁻^1^, whilst performance level 5 individuals (n = 11) averaged 61.4 ± 3.4 mL·kg⁻^1^·min⁻^1^, and 3.60 ± 0.20 L·min⁻^1^ respectively. This demonstrates that $\dot{V}$O_2Max_ is typically higher in XCS than cycling, possibly owing to the importance of aerobic capacity in XCS and the aforementioned differences in muscle mass recruitment [143, 144]. Despite Decroix et al. [142] employing similar procedures to this study, their definitions for performance level were broader than those incorporated in this study. Performance level 4 individuals were described as ‘highly trained, well trained, endurance trained and highly trained competitive’ more aligned with the descriptors of PCF tier 3 in this study, whilst performance level 5 individuals were simply broadly described as ‘professional cyclists’, more aligned with the descriptors combining PCF tier 4 and 5 in this study. These discrepancies in performance definitions, make comparisons across sports challenging. In a study of ‘world-class’ cyclists, males recorded $\dot{V}$O_2Max_ values of 71.3 ± 4.2 mL·kg⁻^1^·min⁻^1^ and 5.02 ± 0.36 L·min⁻^1^ [145], demonstrating an apparent alignment to values reported in XCS for PCF tier 4. Whilst these $\dot{V}$O_2Max_ values for the cyclist population may have been classified as ‘elite’ (PCF tier 4) using benchmarks predating the PCF [146], the group included ‘world class’, such as World Championship podium finishers, more aligned with PCF tier 5, This suggests XCS athletes possess higher $\dot{V}$O_2Max_ values. In the same study [145], ‘amateur competitive cyclists’ (PCF tier 3) showed very similar $\dot{V}$O_2Max_ values (69.5 ± 5.0 mL·kg⁻^1^·min⁻^1^, 4.86 ± 0.43 L·min⁻^1^), to those of the PCF tier 5 group. While limited to a single representative study, this example suggests that $\dot{V}$O_2Max_ variability in XCS is broader compared to more nuanced differences between PCF tiers in male cycling.

In addition, Sandbakk et al. [4] compared a range of physiological variables between more homogenous populations, world class (PCF Tier 5) and nationally competing (PCF Tier 3) athletes. Results demonstrated clear relationships between $\dot{V}$O_2Peak_ and performance when comparing groups (Tier 3 vs. Tier 5) (*p* < 0.05), but no significant relationships within-groups (*p* > 0.05). Supporting this, Tønnessen et al. (2015) [92] found statistically non-significant differences between mean $\dot{V}$O_2Max_ of medallists and non-medallists in both distance and sprint XCS events, for males and females, giving brief mention to working economy and fractional utilisation of $\dot{V}$O_2Max_ as being other factors determining performance. Differences in distance events were described as ‘moderate’, whilst differences for sprint events were described as ‘trivial’. Pooled data from the appraised literature in this study demonstrate the importance of $\dot{V}$O_2Max_ to distinguish differences in PCF tiers, even at tiers 4 and 5, but further empirical evidence may be required in XCS to be confident of this finding [92].

Individual athlete case studies were excluded owing to the lack of central tendency statistics necessary for subgroup regression analyses undertaken in this study [147–149]. Formenti et al. [147], did not report $\dot{V}$O_2Max_ values and would subsequently have been excluded under criterion 5. Hladnick et al. [148] reported a $\dot{V}$O_2Max_ value of 63.2 mL·kg^−1^·min^−1^ in a Slovenian national cross-country team member (PCF tier 4), whilst Talsnes et al. [149] reported $\dot{V}$O_2Max_ values of 5.59 L·min^−1^ and 79.9 mL·min^−1^·kg^−1^ in an athlete classified as PCF tier 5. Both of these case studies used the G3 skate technique. Furthermore, Norwegian Bjørn Dæhlie’s reputed $\dot{V}$O_2Max_ has been reported as ~ 96.0 mL·min^−1^, but owing to the non-peer reviewed sources of this value, these data were also not included.

The observed trend of $\dot{V}$O_2Max_ increasing with PCF tier was constant across both relative (Fig. 7A) and absolute measurements (Fig. 7C) in males. However, whilst females also demonstrated this trend in relative measures (Fig. 7A), this was not as obviously consistent in absolute measures (Fig. 7C). This supports the assertion that body mass of international (PCF tier 4) and world-class (PCF tier 5) female athletes is a contributory factor to performance success [150–152]. A caveat to this point is the number of studies [22, 58, 81] and relatively low combined sample size (n = 24), contributing to the PCF tier 3 subgroup mean. Johansen et al. [58] reported values of three national level female athletes at three distinct time points, combined here to produce a sample size of 9. One participant reported an absolute value > 5.00 L·min^−1^, during their second repeat assessment, elevating the average for this study. Despite the study explicitly reporting their participants as being ‘national level’ (PCF tier 3) it is possible this individual transitioned to international standard during the course of the study, which may have resulted in an inflated PCF tier 3 subgroup average.

The findings of this study also highlighted sex differences between male and female athletes. Female relative $\dot{V}$O_2Max_ differences ranged between 13–17% lower, whilst absolute values were between 22 and 33% lower. The relative values recorded in this study agree with a recent study by Solli et al. [152], reporting females as typically demonstrating between 10 and 27% lower values. However, whilst overlapping, the range of absolute values reported in this study are slightly lower than those of Solli et al. [152] who reported a range between 30 and 63%. This slight divergence is possibly explained by the present study only focussing on running as a testing modality and only investigating cross-country skiers. Solli et al. [152] meanwhile, included ‘$\dot{V}$O_2Peak_’ values from protocols including diagonal stride and double polling, as well as the G2 and G3 skate techniques. Furthermore, Solli et al. [152] included data for a broader range of Nordic skiers, such as biathletes and ski mountaineers, as well as cross-country skiing athletes, potentially impacting direct transferability across these studies.

### Practical Application

For academic assessments of XCS studies, the amended PCF (Table 1) may serve as a guide for future researchers [23]. Additionally, The findings of this study may inform various performance-related practices.

While long-term athletic development is complex and often involves exceptions (e.g., late vs. early developers), endurance performance maturation remains critical for XCS success. The PCF tiers align with key athletic development stages, ranging from recreationally active tier 1 individuals to world-class medallists at tier 5. Coaches may find the reported values valuable for evaluating their athletes' performance potential. For instance, an athlete with a significantly lower-than-expected $\dot{V}$O_2Max_ for their development phase is unlikely to achieve competitiveness without substantial progress, which may be challenging to attain efficiently. Thus, the $\dot{V}$O_2Max_ benchmarks outlined by PCF tier may assist in talent identification, talent transfer from other sports, and athlete selection criteria when used alongside other metrics [153, 154].

### Limitations

This study is not without limitations. Firstly, insufficient data were identified across sufficient PCF tiers to meaningfully analyse XCS specific techniques. Despite this, previous research suggests $\dot{V}$O_2Max_ values between running and DS skiing to be comparable [136]. Running is still a test modality widely used within XCS due to its practical advantages over skiing techniques (i.e. ski treadmills are very expensive). Therefore, the findings in this study remain practically relevant. Secondly, to broaden the inclusion of data, this study included all $\dot{V}$O_2Max_ data irrespective of calculation method, potentially influencing the comparability of studies [106, 115]. Finally, this study did not find sufficient data to allow segregation of data by distance or sprint categories, and therefore the findings of this study are representative of overall XCS performance, with further specific consideration of sprint and distance demands required in the future.

### Future Directions

Comparable to many areas of sports science [155, 156], females are currently under-represented in XCS research (see Sect. "Descriptive Study Details"). Future studies should consider increased female representation, or exclusive female representation to address knowledge gaps. Although outside the scope of this review, other populations, such as para-athletes, are also likely to be under-represented. Consideration as to the most effective techniques to assess para-athlete performance may be considered by future researchers.

This review demonstrates some considerations that need to be given to PCF scoring in XCS, such as the alignment of the FIS point system with the PCF. However, as FIS are changeable from year to year, the points of those in 20th and 300th position will continually fluctuate. As such, the numbers in this study are presented as a guide and may require future updates, or discipline specific considerations.

Most XCS research has been conducted using running. This is unsurprising, owing to the later development of treadmills that safely support roller-skiing, and the wealth of data that exists for running tests. Transitioning to more externally valid methods of assessment, such as roller-skiing, has been debated [80, 136]. Nevertheless, given the continuing increasing accessibility of alternatives (e.g. Double Poling Ski Ergometers), the number of studies utilising “ski-specific” performance assessments are likely to increase. Future research should continue to investigate these XCS specific movements to assess sport-specific applications.

## Conclusions

Re-examining previous data suggests the current heterogeneity even within the international standard (elite and world-class) XCS athletes may explain some of the emphasis placed on the importance of $\dot{V}$O_2Max_ throughout XCS research. Nevertheless, this study provides clear evidence of the importance of $\dot{V}$O_2Max_ to XCS performance, often a differentiating factor between performance tiers and therefore should be a particular focus of those working with and developing XCS athletes.

## Data Availability

Data available on request to the corresponding author.

## References

[1] Holmberg HC. The elite cross-country skier provides unique insights into human exercise physiology. Scand J Med Sci Sports. 2015. 10.1111/sms.12601. 26589123 10.1111/sms.12601

[2] Stöggl T, Pellegrini B, Holmberg HC. Pacing and predictors of performance during cross-country skiing races: a systematic review. J Sport Health Sci. 2018. 10.1016/j.jshs.2018.09.005. 30450246 10.1016/j.jshs.2018.09.005 PMC6234023

[3] Hébert-Losier K, Zinner C, Platt S, Stöggl T, Holmberg HC. Factors that influence the performance of elite sprint cross-country skiers. Sports Med. 2017. 10.1007/s40279-016-0573-2. 27334280 10.1007/s40279-016-0573-2 PMC5266777

[4] Sandbakk Ø, Holmberg HC, Leirdal S, Ettema G. The physiology of world-class sprint skiers. Scand J Med Sci Sports. 2011. 10.1111/j.1600-0838.2010.01117.x. 20500558 10.1111/j.1600-0838.2010.01117.x

[5] Talsnes RK, Brattebø JM, Berdal T, Seeberg T, Skovereng K, Losnegard T, et al. Performance-determining variables of a simulated sprint cross-country skiing competition. Int J Sports Physiol Perform. 2023. 10.1123/ijspp.2023-0268. 37931616 10.1123/ijspp.2023-0268

[6] Vesterinen V, Mikkola J, Nummela A, Hynynen E, Häkkinen K. Fatigue in a simulated cross-country skiing sprint competition. J Sports Sci. 2009. 10.1080/02640410903081860. 19847690 10.1080/02640410903081860

[7] Joyner MJ, Coyle EF. Endurance exercise performance: the physiology of champions. J Physiol. 2008. 10.1113/jphysiol.2007.143834. 17901124 10.1113/jphysiol.2007.143834 PMC2375555

[8] Lundgren KM, Karlsen T, Sandbakk Ø, James PE, Tjønna AE. Sport-specific physiological adaptations in highly trained endurance athletes. Med Sci Sports Exerc. 2015. 10.1249/mss.0000000000000634. 25668407 10.1249/MSS.0000000000000634

[9] Sandbakk Ø, Holmberg HC. A reappraisal of success factors for Olympic cross-country skiing. Int J Sports Physiol Perform. 2014. 10.1123/ijspp.2013-0373. 24088346 10.1123/ijspp.2013-0373

[10] Sandbakk Ø, Hegge AM, Losnegard T, Skattebo Ø, Tønnessen E, Holmberg HC. The physiological capacity of the world’s highest ranked female cross-country skiers. Med Sci Sports Exerc. 2016. 10.1249/mss.0000000000000862. 26741124 10.1249/MSS.0000000000000862 PMC5642331

[11] Ingjer F. Maximal oxygen uptake as a predictor of performance ability in women and men elite cross-country skiers. Scand J Med Sci Sports. 1991. 10.1111/j.1600-0838.1991.tb00267.x.

[12] Mahood NV, Kenefick RW, Kertzer R, Quinn TJ. Physiological determinants of cross-country ski racing performance. Med Sci Sports Exerc. 2001. 10.1097/00005768-200108000-00020. 11474341 10.1097/00005768-200108000-00020

[13] Carlsson M, Carlsson T, Knutsson M, Malm C, Tonkonogi M. Oxygen uptake at different intensities and sub-techniques predicts sprint performance in elite male cross-country skiers. Eur J Appl Physiol. 2014. 10.1007/s00421-014-2980-0. 25138966 10.1007/s00421-014-2980-0

[14] Carlsson M, Carlsson T, Wedholm L, Nilsson M, Malm C, Tonkonogi M. Physiological demands of competitive sprint and distance performance in elite female cross-country skiing. J Strength Cond Res. 2016. 10.1519/jsc.0000000000001327. 26808846 10.1519/JSC.0000000000001327

[15] Talsnes RK, Solli GS, Kocbach J, Torvik P, Sandbakk Ø. Laboratory- and field-based performance predictions in cross-country skiing and roller-skiing. PLoS ONE. 2021. 10.1371/journal.pone.0256662. 34428258 10.1371/journal.pone.0256662 PMC8384222

[16] Bassett DR Jr, Howley ET. Limiting factors for maximum oxygen uptake and determinants of endurance performance. Med Sci Sports Exerc. 2000. 10.1097/00005768-200001000-00012. 10647532 10.1097/00005768-200001000-00012

[17] Costill DL. The relationship between selected physiological variables and distance running performance. J Sports Med Phys Fitness. 1967;7(2):61–6. 6074901

[18] Legaz-Arrese A, Munguía-Izquierdo D, Nuviala A, Serveto-Galindo O, Moliner Urdiales D, Reverter Masía J. Average VO2max as a function of running performances on different distances. Sci Sports. 2007. 10.1016/j.scispo.2006.01.008.

[19] McLaughlin JE, Howley ET, Bassett DR Jr, Thompson DL, Fitzhugh EC. Test of the classic model for predicting endurance running performance. Med Sci Sports Exerc. 2010. 10.1249/mss.0b013e3181c0669d. 19997010 10.1249/MSS.0b013e3181c0669d

[20] Saltin B, Åstrand PO. Maximal oxygen uptake in athletes. J Appl Physiol. 1967. 10.1152/jappl.1967.23.3.353. 6047957 10.1152/jappl.1967.23.3.353

[21] Sjödin B, Svedenhag J. Applied physiology of marathon running. Sports Med. 1985. 10.2165/00007256-198502020-00002. 3890068 10.2165/00007256-198502020-00002

[22] Bergh U. The influence of body mass in cross-country skiing. Med Sci Sports Exerc. 1987;19(4):324–31. 3657480

[23] McKay AKA, Stellingwerff T, Smith ES, Martin DT, Mujika I, Goosey-Tolfrey VL, et al. Defining training and performance caliber: a participant classification framework. Int J Sports Physiol Perform. 2022. 10.1123/ijspp.2021-0451. 34965513 10.1123/ijspp.2021-0451

[24] Page MJ, McKenzie JE, Bossuyt PM, Boutron I, Hoffmann TC, Mulrow CD, et al. The PRISMA 2020 statement: an updated guideline for reporting systematic reviews. BMJ. 2021. 10.1136/bmj.n71. 33782057 10.1136/bmj.n71 PMC8005924

[25] International Ski and Snowboard Federation. Cross-country skiing FIS points lists 2024. https://www.fis-ski.com/DB/cross-country/fis-points-lists.html?mi=menu-fis-points.

[26] Karlsson O, Laaksonen MS, McGawley K. Training and illness characteristics of cross-country skiers transitioning from junior to senior level. PLoS One. 2021. 10.1371/journal.pone.0250088 10.1371/journal.pone.0250088 PMC8121355 33989314

[27] Cochrane. Cochrane handbook for systematic reviews of interventions. 2024. www.training.cochrane.org/handbook.

[28] Knapp G, Hartung J. Improved tests for a random effects meta-regression with a single covariate. Stat Med. 2003. 10.1002/sim.1482. 12939780 10.1002/sim.1482

[29] IntHout J, Ioannidis JPA, Rovers MM, Goeman JJ. Plea for routinely presenting prediction intervals in meta-analysis. BMJ Open. 2016. 10.1136/bmjopen-2015-010247. 27406637 10.1136/bmjopen-2015-010247 PMC4947751

[30] Higgins JP. Commentary: heterogeneity in meta-analysis should be expected and appropriately quantified. Int J Epidemiol. 2008. 10.1093/ije/dyn204. 18832388 10.1093/ije/dyn204

[31] Alsobrook NG, Heil DP. Upper body power as a determinant of classical cross-country ski performance. Eur J Appl Physiol. 2009. 10.1007/s00421-008-0943-z. 19039602 10.1007/s00421-008-0943-z

[32] Andersson EP, McGawley K. A comparison between different methods of estimating anaerobic energy production. Front Physiol. 2018. 10.3389/fphys.2018.00082. 29472871 10.3389/fphys.2018.00082 PMC5809502

[33] Andersson E, Björklund G, Holmberg HC, Ortenblad N. Energy system contributions and determinants of performance in sprint cross-country skiing. Scand J Med Sci Sports. 2017. 10.1111/sms.12666. 26923666 10.1111/sms.12666

[34] Andersson E, Holmberg HC, Ortenblad N, Björklund G. Metabolic responses and pacing strategies during successive sprint skiing time trials. Med Sci Sports Exerc. 2016. 10.1249/mss.0000000000001037. 27414686 10.1249/MSS.0000000000001037

[35] Berg J, Undebakke V, Rasch-Halvorsen Ø, Åkerøy L, Sandbakk Ø, Tjønna AE. Comparison of mitochondrial respiration in *M. triceps* brachii and *M. vastus* lateralis between elite cross-country skiers and physically active controls. Front Physiol. 2019. 10.3389/fphys.2019.00365. 31024334 10.3389/fphys.2019.00365 PMC6461012

[36] Bilodeau B, Roy B, Boulay MR. Upper-body testing of cross-country skiers. Med Sci Sports Exerc. 1995;27(11):1557–62. 8587493

[37] Binek M, Drzazga Z, Socha T, Pokora I. Do gender differences in skin temperature of lower limbs following exercise test in male and female cross-country skiers exist? J Therm Anal Calorim. 2022. 10.1007/s10973-021-11055-z.

[38] Björklund G, Laaksonen MS, Holmberg HC. Blood lactate recovery and respiratory responses during diagonal skiing of variable intensity. Eur J Sport Sci. 2011. 10.1080/17461391.2010.521580.

[39] Bolger CM, Kocbach J, Hegge AM, Sandbakk Ø. Speed and heart-rate profiles in skating and classical cross-country-skiing competitions. Int J Sports Physiol Perform. 2015. 10.1123/ijspp.2014-0335. 25671845 10.1123/ijspp.2014-0335

[40] Bucher E, Millet GP, Wehrlin JP, Steiner T. Test–retest reliability of ski-specific aerobic, sprint, and neuromuscular performance tests in highly trained cross-country skiers. Scand J Med Sci Sports. 2023. 10.1111/sms.14473. 37635277 10.1111/sms.14473

[41] Bucher E, Sandbakk O, Donath L, Roth R, Zahner L, Faude O. Exercise-induced trunk fatigue decreases double poling performance in well-trained cross-country skiers. Eur J Appl Physiol. 2018. 10.1007/s00421-018-3938-4. 30006669 10.1007/s00421-018-3938-4

[42] Carlsson M, Carlsson T, Hammarström D, Tiivel T, Malm C, Tonkonogi M. Validation of physiological tests in relation to competitive performances in elite male distance cross-country skiing. J Strength Cond Res. 2012. 10.1519/jsc.0b013e318231a799. 22614140 10.1519/JSC.0b013e318231a799

[43] Carlsson T, Carlsson M, Hammarström D, Rønnestad BR, Malm CB, Tonkonogi M. Optimal VO2max-to-mass ratio for predicting 15 km performance among elite male cross-country skiers. Open Access J Sports Med. 2015. 10.2147/oajsm.s93174. 26719730 10.2147/OAJSM.S93174 PMC4689292

[44] Danielsen J, Sandbakk Ø, Holmberg HC, Ettema G. Mechanical energy and propulsion in ergometer double poling by cross-country skiers. Med Sci Sports Exerc. 2015. 10.1249/mss.0000000000000723. 26110695 10.1249/MSS.0000000000000723

[45] Drzazga Z, Binek M, Pokora I, Sadowska-Krępa E. A preliminary study on infrared thermal imaging of cross-country skiers and swimmers subjected to endurance exercise. J Therm Anal Calorim. 2018. 10.1007/s10973-018-7311-y.

[46] Evertsen F, Medbø JI, Jebens E, Gjovaag TF. Effect of training on the activity of five muscle enzymes studied on elite cross-country skiers. Acta Physiol Scand. 1999. 10.1046/j.1365-201x.1999.00607.x. 10606827 10.1046/j.1365-201x.1999.00607.x

[47] Fabre N, Passelergue P, Bouvard M, Perrey S. Comparison of heart rate deflection and ventilatory threshold during a field cross-country roller-skiing test. J Strength Cond Res. 2008. 10.1519/jsc.0b013e3181874ae9. 18978612 10.1519/JSC.0b013e3181874ae9

[48] Gejl KD, Ørtenblad N, Andersson E, Plomgaard P, Holmberg HC, Nielsen J. Local depletion of glycogen with supramaximal exercise in human skeletal muscle fibres. J Physiol. 2017. 10.1113/jp273109. 27689320 10.1113/JP273109 PMC5407966

[49] Grasaas CA, Ettema G, Hegge AM, Skovereng K, Sandbakk O. Changes in technique and efficiency after high-intensity exercise in cross-country skiers. Int J Sports Physiol Perform. 2014. 10.1123/ijspp.2013-0344. 10.1123/ijspp.2013-0344 23982869

[50] Haymes EM, Dickinson AL. Characteristics of elite male and female ski racers. Med Sci Sports Exerc. 1980;12(3):153–8. 7402049

[51] Hegge AM, Ettema G, de Koning JJ, Rognstad AB, Hoset M, Sandbakk Ø. The effects of the arm swing on biomechanical and physiological aspects of roller ski skating. Hum Mov Sci. 2014. 10.1016/j.humov.2014.05.001. 24893335 10.1016/j.humov.2014.05.001

[52] Hegge AM, Myhre K, Welde B, Holmberg HC, Sandbakk Ø. Are gender differences in upper-body power generated by elite cross-country skiers augmented by increasing the intensity of exercise? PLoS ONE. 2015. 10.1371/journal.pone.0127509. 26000713 10.1371/journal.pone.0127509 PMC4441444

[53] Hegge A, Bucher E, Ettema G, Faude O, Holmberg HC, Sandbakk Ø, et al. Gender differences in power production, energetic capacity and efficiency of elite cross-country skiers during whole-body, upper-body, and arm poling. Eur J Appl Physiol. 2016. 10.1007/s00421-015-3281-y. 26476546 10.1007/s00421-015-3281-y

[54] Hoff J, Helgerud J, Wisløff U. Maximal strength training improves work economy in trained female cross-country skiers. Med Sci Sports Exerc. 1999. 10.1097/00005768-199906000-00016. 10378915 10.1097/00005768-199906000-00016

[55] Holmberg HC, Rosdahl H, Svedenhag J. Lung function, arterial saturation and oxygen uptake in elite cross-country skiers: Influence of exercise mode. Scand J Med Sci Sports. 2007. 10.1111/j.1600-0838.2006.00592.x. 17040487 10.1111/j.1600-0838.2006.00592.x

[56] Ingjer F, Myhre K. Physiological effects of altitude training on elite male cross-country skiers. J Sports Sci. 1992. 10.1080/02640419208729905. 1556777 10.1080/02640419208729905

[57] Johansen J-M, Eriksen S, Sunde A, Slettemeås ØB, Helgerud J, Støren Ø. Improving utilization of maximal oxygen uptake and work economy in recreational cross-country skiers with high-intensity double-poling intervals. Int J Sports Physiol Perform. 2021. 10.1123/ijspp.2019-0689. 32604071 10.1123/ijspp.2019-0689

[58] Johansen JM, Sunde A, Helgerud J, Gjerløw LE, Støren Ø. Effects of individual changes in training distribution on maximal aerobic capacity in well-trained cross-country skiers: a follow-up study. Front Physiol. 2021. 10.3389/fphys.2021.675273. 34262473 10.3389/fphys.2021.675273 PMC8273762

[59] Klusiewicz A, Cempa W. Verification of anaerobic threshold indicators for cross-country skiers in natural conditions. Polish J Sport Tour. 2011. 10.2478/v10197-011-0025-3.

[60] Kravitz L, Robergs RA, Heyward VH, Wagner DR, Powers K. Exercise mode and gender comparisons of energy expenditure at self-selected intensities. Med Sci Sports Exerc. 1997. 10.1097/00005768-199708000-00007. 9268959 10.1097/00005768-199708000-00007

[61] Lindinger SJ, Stoeggl T, Mueller E, Holmberg HC. Control of speed during the double poling technique performed by elite cross-country skiers. Med Sci Sports Exerc. 2009. 10.1249/mss.0b013e318184f436. 19092686 10.1249/MSS.0b013e318184f436

[62] Losnegard T, Mikkelsen K, Ronnestad BR, Hallen J, Rud B, Raastad T. The effect of heavy strength training on muscle mass and physical performance in elite cross-country skiers. Scand J Med Sci Sports. 2011. 10.1111/j.1600-0838.2009.01074.x. 20136751 10.1111/j.1600-0838.2009.01074.x

[63] Losnegard T, Myklebust H, Hallén J. No differences in O2-cost between V1 and V2 skating techniques during treadmill roller skiing at moderate to steep inclines. J Strength Cond Res. 2012. 10.1519/jsc.0b013e318231a69e. 22516907 10.1519/JSC.0b013e318231a69e

[64] Mognoni P, Rossi G, Gastaldelli F, Canclini A, Cotelli F. Heart rate profiles and energy cost of locomotion during cross-country skiing races. Eur J Appl Physiol. 2001. 10.1007/s004210100432. 11513322 10.1007/s004210100432

[65] Myakinchenko EB, Heil DP, Kriuchkov AS, Feofilaktov VV, Kuzmichev VA, Adodin NV. Physiological profiles and training loads of international level male and female cross-country skiers and biathletes. Sci Sports. 2022. 10.1016/j.scispo.2021.09.004.

[66] Myakinchenko EB, Kriuchkov AS, Adodin NV, Feofilaktov V. The annual periodization of training volumes of international-level cross-country skiers and biathletes. Int J Sports Physiol Perform. 2020. 10.1123/ijspp.2019-0220. 32820140 10.1123/ijspp.2019-0220

[67] Mygind E, Larsson B, Klausen T. Evaluation of a specific test in cross-country skiing. J Sports Sci. 1991. 10.1080/02640419108729887. 1960796 10.1080/02640419108729887

[68] Ng AV, Demment RB, Bassett DR, Bussan MJ, Clark RR, Kuta JM, et al. Characteristics and performance of male citizen cross-country ski racers. Int J Sports Med. 1988. 10.1055/s-2007-1025007. 3410626 10.1055/s-2007-1025007

[69] Nybäck L, Glännerud C, Larsson G, Weitzberg E, Shannon OM, McGawley K. Physiological and performance effects of nitrate supplementation during roller-skiing in normoxia and normobaric hypoxia. Nitric Oxide. 2017. 10.1016/j.niox.2017.08.001. 28782598 10.1016/j.niox.2017.08.001

[70] Østerås S, Welde B, Danielsen J, Van Den Tillaar R, Ettema G, Sandbakk O. Contribution of upper-body strength, body composition, and maximal oxygen uptake to predict double poling power and overall performance in female cross-country skiers. J Strength Cond Res. 2016. 10.1519/jsc.0000000000001345. 26817743 10.1519/JSC.0000000000001345

[71] Rud B, Oygard E, Dahl EB, Paulsen G, Losnegard T. The effect of resistance exercise priming in the morning on afternoon sprint cross-country skiing performance. Int J Sports Physiol Perform. 2021. 10.1123/ijspp.2020-0881. 34021095 10.1123/ijspp.2020-0881

[72] Sandbakk O, Ettema G, Holmberg HC. Gender differences in endurance performance by elite cross-country skiers are influenced by the contribution from poling. Scand J Med Sci Sports. 2014. 10.1111/j.1600-0838.2012.01482.x. 22621157 10.1111/j.1600-0838.2012.01482.x

[73] Sandbakk Ø, Ettema G, Holmberg HC. The physiological and biomechanical contributions of poling to roller ski skating. Eur J Appl Physiol. 2013. 10.1007/s00421-013-2629-4. 23543069 10.1007/s00421-013-2629-4

[74] Sandbakk Ø, Ettema G, Leirdal S, Holmberg HC. Gender differences in the physiological responses and kinematic behaviour of elite sprint cross-country skiers. Eur J Appl Physiol. 2012. 10.1007/s00421-011-2063-4. 21748369 10.1007/s00421-011-2063-4 PMC3276766

[75] Sandbakk O, Ettema G, Leirdal S, Jakobsen V, Holmberg HC. Analysis of a sprint ski race and associated laboratory determinants of world-class performance. Eur J Appl Physiol. 2011. 10.1007/s00421-010-1719-9. 21079989 10.1007/s00421-010-1719-9 PMC3092926

[76] Sandbakk Ø, Skålvik TF, Spencer M, van Beekvelt M, Welde B, Hegge AM, et al. The physiological responses to repeated upper-body sprint exercise in highly trained athletes. Eur J Appl Physiol. 2015. 10.1007/s00421-015-3128-6. 25677383 10.1007/s00421-015-3128-6

[77] Sandbakk SB, Sandbakk Ø, Peacock O, James P, Welde B, Stokes K, et al. Effects of acute supplementation of L-arginine and nitrate on endurance and sprint performance in elite athletes. Nitric Oxide. 2015. 10.1016/j.niox.2014.10.006. 25445632 10.1016/j.niox.2014.10.006

[78] Schmitt L, Willis SJ, Coulmy N, Millet GP. Effects of different training intensity distributions between elite cross-country skiers and Nordic-combined athletes during live high-train low. Front Physiol. 2018. 10.3389/fphys.2018.00932. 30072913 10.3389/fphys.2018.00932 PMC6060253

[79] Skattebo O, Hallen J, Ronnestad BR, Losnegard T. Upper body heavy strength training does not affect performance in junior female cross-country skiers. Scand J Med Sci Sports. 2016. 10.1111/sms.12517. 26146761 10.1111/sms.12517

[80] Skattebo O, Losnegard T, Stadheim HK. Double-poling physiology and kinematics of elite cross-country skiers: specialized long-distance versus all-round skiers. Int J Sports Physiol Perform. 2019. 10.1123/ijspp.2018-0471. 30840518 10.1123/ijspp.2018-0471

[81] Solli GS, Haugnes P, Kocbach J, van den Tillaar R, Torvik PO, Sandbakk O. The effects of a short specific versus a long traditional warm-up on time-trial performance in cross-country skiing sprint. Int J Sports Physiol Perform. 2020. 10.1123/ijspp.2019-0618. 32182587 10.1123/ijspp.2019-0618

[82] Solli GS, Kocbach J, Seeberg TM, Tjonnas J, Rindal OMH, Haugnes P, et al. Sex-based differences in speed, sub-technique selection, and kinematic patterns during low- and high-intensity training for classical cross-country skiing. PLoS ONE. 2018. 10.1371/journal.pone.0207195. 30440017 10.1371/journal.pone.0207195 PMC6237352

[83] Stadheim HK, Kvamme B, Olsen R, Drevon CA, Ivy JL, Jensen J. Caffeine increases performance in cross-country double-poling time trial exercise. Med Sci Sports Exerc. 2013. 10.1249/mss.0b013e3182967948. 23591294 10.1249/MSS.0b013e3182967948

[84] Stadheim HK, Nossum EM, Olsen R, Spencer M, Jensen J. Caffeine improves performance in double poling during acute exposure to 2000-m altitude. J Appl Physiol. 2015. 10.1152/japplphysiol.00509.2015. 26494444 10.1152/japplphysiol.00509.2015

[85] Stöggl T, Enqvist J, Müller E, Holmberg HC. Relationships between body composition, body dimensions, and peak speed in cross-country sprint skiing. J Sports Sci. 2010. 10.1080/02640410903414160. 20391090 10.1080/02640410903414160

[86] Stöggl T, Lindinger S, Müller E. Analysis of a simulated sprint competition in classical cross-country skiing. Scand J Med Sci Sports. 2007. 10.1111/j.1600-0838.2006.00589.x. 16911588 10.1111/j.1600-0838.2006.00589.x

[87] Stöggl T, Müller E, Ainegren M, Holmberg HC. General strength and kinetics: fundamental to sprinting faster in cross-country skiing? Scand J Med Sci Sports. 2011. 10.1111/j.1600-0838.2009.01078.x. 20492588 10.1111/j.1600-0838.2009.01078.x

[88] Sunde A, Johansen JM, Gjora M, Paulsen G, Braten M, Helgerud J, et al. Stronger is better: the impact of upper body strength in double poling performance. Front Physiol. 2019. 10.3389/fphys.2019.01091. 31507453 10.3389/fphys.2019.01091 PMC6716506

[89] Sylta Ø, Tønnessen E, Seiler S. From heart-rate data to training quantification: a comparison of 3 methods of training-intensity analysis. Int J Sports Physiol Perform. 2014. 10.1123/ijspp.2013-0298. 24408353 10.1123/IJSPP.2013-0298

[90] Sylta Ø, Tønnessen E, Seiler S. Do elite endurance athletes report their training accurately? Int J Sports Physiol Perform. 2014. 10.1123/ijspp.2013-0203. 23921186 10.1123/ijspp.2013-0203

[91] Talsnes RK, Solli GS, Kocbach J, Torvik PO, Sandbakk O. Laboratory- and field-based performance predictions in cross-country skiing and roller skiing. PLoS ONE. 2021. 10.1371/journal.pone.0256662. 34428258 10.1371/journal.pone.0256662 PMC8384222

[92] Tønnessen E, Haugen TA, Hem E, Leirstein S, Seiler S. Maximal aerobic capacity in the Winter-Olympics endurance disciplines: Olympic-medal benchmarks for the time period 1990–2013. Int J Sports Physiol Perform. 2015. 10.1123/ijspp.2014-0431. 25611016 10.1123/ijspp.2014-0431

[93] Tortu E, Ouergui I, Deliceoğlu G, Keleş A, Ulupinar E. Aerobic capacity and respiratory indices of junior cross-country skiers and biathletes during incremental exercise testing. Sci Rep. 2024. 10.1038/s41598-024-73365-0. 39333270 10.1038/s41598-024-73365-0 PMC11437039

[94] Torvik PØ, van den Tillaar R, Iversen G. Does the order of submaximal lactate threshold and maximal oxygen uptake testing influence test outcomes? Sports. 2020. 10.3390/sports8060075. 32466372 10.3390/sports8060075 PMC7353634

[95] Torvik PO, Persson J, van den Tillaar R. The effects of sub-technique and pole length on classic roller skiing performance and physiological responses at steep uphill inclination. J Hum Kinet. 2021. 10.2478/hukin-2021-0014. 34168695 10.2478/hukin-2021-0014 PMC8008307

[96] Undebakke V, Berg J, Tjønna AE, Sandbakk O. Comparison of physiological and perceptual responses to upper-, lower-, and whole-body exercise in elite cross-country skiers. J Strength Cond Res. 2019. 10.1519/jsc.0000000000003078. 30741871 10.1519/JSC.0000000000003078

[97] Verges S, Flore P, Favre-Juvin A. Blood lactate concentrating/heart rate relationship: laboratory running test vs. field roller skiing test. Int J Sports Med. 2003. 10.1055/s-2003-41176. 12905094 10.1055/s-2003-41176

[98] Verges S, Flore P, Laplaud D, Guinot M, Favre-Juvin A. Laboratory running test vs. field roller skiing test in cross-country skiers: a longitudinal study. Int J Sports Med. 2006. 10.1055/s-2005-865664. 16572374 10.1055/s-2005-865664

[99] Walther J, Haugen T, Solli GS, Tønnessen E, Sandbakk O. From juniors to seniors: changes in training characteristics and aerobic power in 17 world-class cross-country skiers. Front Physiol. 2023. 10.3389/fphys.2023.1288606. 38054044 10.3389/fphys.2023.1288606 PMC10694351

[100] Wang JC, Yim KT, Choi YC. Effects of LHTH training at 1600 m on exercise performance, complete blood count and erythropoietin: a case study of South Korean elite male cross-country skiers. J Men’s Health. 2022. 10.31083/j.jomh1809187.

[101] Wisløff U, Helgerud J, Støylen A, Ellingsen Ø. Atrioventricular plane displacement in female endurance athletes. Med Sci Sports Exerc. 2001. 10.1097/00005768-200109000-00013. 11528339 10.1097/00005768-200109000-00013

[102] Zhu Y, Wang Z, Li R, Li Y, Bai P, Gao W, et al. Skiing economy and kinematic during a field double poling roller skiing among novice and experienced cross-country skiers. Sci Rep. 2024. 10.1038/s41598-024-57719-2. 38528144 10.1038/s41598-024-57719-2 PMC10963750

[103] Myklebust H, Losnegard T, Hallén J. Differences in V1 and V2 ski skating techniques described by accelerometers. Scand J Med Sci Sports. 2014. 10.1111/sms.12106. 23957331 10.1111/sms.12106

[104] Pellegrini B, Sandbakk Ø, Stöggl T, Supej M, Ørtenblad N, Schürer A, et al. Methodological guidelines designed to improve the quality of research on cross-country skiing. J Sci Sport Exerc. 2021. 10.1007/s42978-021-00112-6.

[105] Holsbrekken E, Gløersen Ø, Lund-Hansen M, Losnegard T. Competitive cross-country skiers have longer time to exhaustion than recreational cross-country skiers during intermittent work intervals normalized to their maximal aerobic power. Int J Sports Physiol Perform. 2023. 10.1123/ijspp.2022-0487. 37567577 10.1123/ijspp.2022-0487

[106] Staunton CA, Andersson EP, Skovereng K, Björklund G. Heart rate does not accurately predict metabolic intensity during variable-intensity roller skiing or cycling. Int J Sports Physiol Perform. 2022. 10.1123/ijspp.2022-0114. 36343622 10.1123/ijspp.2022-0114

[107] Hill AV, Lupton H. Muscular exercise, lactic acid, and the supply and utilization of oxygen. QJM An Int J Med. 1923. 10.1098/rspb.1924.0048.

[108] Midgley AW, Carroll S, Marchant D, McNaughton LR, Siegler J. Evaluation of true maximal oxygen uptake based on a novel set of standardized criteria. Appl Physiol Nutr Metab. 2009. 10.1139/h08-146. 19370041 10.1139/H08-146

[109] Poole DC, Wilkerson DP, Jones AM. Validity of criteria for establishing maximal O2 uptake during ramp exercise tests. Eur J Appl Physiol. 2008. 10.1007/s00421-007-0596-3. 17968581 10.1007/s00421-007-0596-3

[110] Poole DC, Jones AM. Measurement of the maximum oxygen uptake o_2_max: o_2_peak is no longer acceptable. J Appl Physiol. 2017. 10.1152/japplphysiol.01063.2016. 28153947 10.1152/japplphysiol.01063.2016

[111] Bowen TS, Cannon DT, Begg G, Baliga V, Witte KK, Rossiter HB. A novel cardiopulmonary exercise test protocol and criterion to determine maximal oxygen uptake in chronic heart failure. J Appl Physiol. 2012. 10.1152/japplphysiol.01416.2011. 22653993 10.1152/japplphysiol.01416.2011 PMC3426168

[112] Whipp BJ. Physiological mechanisms dissociating pulmonary CO2 and O2 exchange dynamics during exercise in humans. Exp Physiol. 2007. 10.1113/expphysiol.2006.034363. 17185348 10.1113/expphysiol.2006.034363

[113] Niemeyer M, Knaier R, Beneke R. The oxygen uptake plateau-a critical review of the frequently misunderstood phenomenon. Sports Med. 2021. 10.1007/s40279-021-01471-4. 33914281 10.1007/s40279-021-01471-4 PMC8363556

[114] Green S, Askew C. o_2peak_ is an acceptable estimate of cardiorespiratory fitness but not o_2max_. J Appl Physiol. 2018. 10.1152/japplphysiol.00850.2017. 29420148 10.1152/japplphysiol.00850.2017

[115] Martin-Rincon M, Calbet JAL. Progress update and challenges on V.O2max testing and interpretation. Front Physiol. 2020. 10.3389/fphys.2020.01070. 33013459 10.3389/fphys.2020.01070 PMC7494971

[116] Azevedo P, Bhammar DM, Babb TG, Bowen TS, Witte KK, Rossiter HB, et al. Commentaries on Viewpoint: o2peak is an acceptable estimate of cardiorespiratory fitness but not o2max. J Appl Physiol. 2018. 10.1152/japplphysiol.00319.2018. 30043694 10.1152/japplphysiol.00319.2018 PMC7528370

[117] Cooper DM. Rethinking Vo2max: right problem, wrong solution (Letter to the Editor regarding Poole and Jones’ “Measurement of the maximum oxygen uptake Vo2max: Vo2peak is no longer acceptable”). J Appl Physiol. 2017. 10.1152/japplphysiol.00396.2017. 28830932 10.1152/japplphysiol.00396.2017

[118] Green S, Askew C. Last word on viewpoint: Vo2peak is an acceptable estimate of cardiorespiratory fitness but not Vo2max. J Appl Physiol. 2018. 10.1152/japplphysiol.00307.2018. 30043696 10.1152/japplphysiol.00307.2018

[119] Pettitt RW, Jamnick NA. Commentary on “measurement of the maximum oxygen uptake Vo2max: Vo2peak is no longer acceptable.” J Appl Physiol. 2017. 10.1152/japplphysiol.00338.2017. 28947630 10.1152/japplphysiol.00338.2017

[120] Poole DC, Jones AM. Reply to Cooper’s letter in reference to: measurement of the maximum oxygen uptake Vo2max: Vo2peak is no longer acceptable. J Appl Physiol. 2017. 10.1152/japplphysiol.00442.2017. 28830933 10.1152/japplphysiol.00442.2017

[121] Poole DC, Jones AM. Reply to Drs. Van Breda et al. J Appl Physiol. 2017. 10.1152/japplphysiol.00231.2017. 28522748 10.1152/japplphysiol.00231.2017

[122] Ainegren M, Carlsson P, Tinnsten M, Laaksonen MS. Skiing economy and efficiency in recreational and elite cross-country skiers. J Strength Cond Res. 2013. 10.1519/jsc.0b013e31824f206c. 22344058 10.1519/JSC.0b013e31824f206c

[123] Andersson EP, Hämberg I, Do Nascimento Salvador PC, McGawley K. Physiological responses and cycle characteristics during double-poling versus diagonal-stride roller-skiing in junior cross-country skiers. Eur J Appl Physiol. 2021. 10.1007/s00421-021-04689-2. 33893836 10.1007/s00421-021-04689-2 PMC8260529

[124] Andersson EP, Noordhof DA, Logdal N. The anaerobic capacity of cross-country skiers: the effect of computational method and skiing sub-technique. Front Sports Act Living. 2020. 10.3389/fspor.2020.00037. 33345029 10.3389/fspor.2020.00037 PMC7739726

[125] Büchel D, Torvik PØ, Lehmann T, Sandbakk Ø, Baumeister J. The mode of endurance exercise influences changes in EEG resting-state graphs among high-level cross-country skiers. Med Sci Sports Exerc. 2023. 10.1249/mss.0000000000003122. 36604783 10.1249/MSS.0000000000003122

[126] Danielsen J, Sandbakk O, McGhie D, Ettema G. The effect of exercise intensity on joint power and dynamics in ergometer double-poling performed by cross-country skiers. Hum Mov Sci. 2018. 10.1016/j.humov.2017.11.010. 29179043 10.1016/j.humov.2017.11.010

[127] Fabre N, Balestreri F, Leonardi A, Schena F. Racing performance and incremental double poling test on treadmill in elite female cross-country skiers. J Strength Cond Res. 2010. 10.1519/jsc.0b013e3181c4d358. 20072060 10.1519/JSC.0b013e3181c4d358

[128] Fabre N, Mourot L, Zoppirolli C, Andersson E, Willis SJ, Holmberg HC. Alterations in aerobic energy expenditure and neuromuscular function during a simulated cross-country skiathlon with the skating technique. Hum Mov Sci. 2015. 10.1016/j.humov.2015.01.014. 25681656 10.1016/j.humov.2015.01.014

[129] Francescato MP, Puntel I. Does a pre-exercise carbohydrate feeding improve a 20-km cross-country ski performance? J Sports Med Phys Fitness. 2006;46(2):248–56. 16823355

[130] Govus A, Marsland F, Martin D, Chapman D. Validity and reliability of an incremental double poling protocol in cross-country skiers. J Hum Sport Exerc. 2015. 10.14198/jhse.2015.103.08.

[131] Hansen LM, Sandbakk Ø, Ettema G, Baumgart JK. Upper- vs. lower-body exercise performance in female and male cross-country skiers. Front Sports Act Living. 2021. 10.3389/fspor.2021.762794. 34993468 10.3389/fspor.2021.762794 PMC8724206

[132] Haugnes P, Kocbach J, Luchsinger H, Ettema G, Sandbakk Ø. The interval-based physiological and mechanical demands of cross-country ski training. Int J Sports Physiol Perform. 2019. 10.1123/ijspp.2018-1007. 30958055 10.1123/ijspp.2018-1007

[133] Hoffman MD, Clifford PS, Watts PB, O’Hagan KP, Mittelstadt SW. Delta efficiency of uphill roller skiing with the double pole and diagonal stride techniques. Can J Appl Physiol. 1995. 10.1139/h95-037. 8563678 10.1139/h95-037

[134] Larsson P, Henriksson-Larsen A. Combined metabolic gas analyser and dGPS analysis of performance in cross-country skiing. J Sports Sci. 2005. 10.1080/02640410400022078. 16195038 10.1080/02640410400022078

[135] Larsson P, Olofsson P, Jakobsson E, Burlin L, Henriksson-Larsen K. Physiological predictors of performance in cross-country skiing from treadmill tests in male and female subjects. Scand J Med Sci Sports. 2002. 10.1034/j.1600-0838.2002.01161.x. 12453161 10.1034/j.1600-0838.2002.01161.x

[136] Losnegard T, Hallén J. Physiological differences between sprint- and distance-specialized cross-country skiers. Int J Sports Physiol Perform. 2014. 10.1123/ijspp.2013-0066. 24155024 10.1123/ijspp.2013-0066

[137] McGawley K, Van Waerbeke C, Westberg K-J, Andersson EP. Maximizing recovery time between knock-out races improves sprint cross-country skiing performance. J Sport Health Sci. 2022. 10.1016/j.jshs.2021.12.004. 34936939 10.1016/j.jshs.2021.12.004 PMC8848028

[138] Sandbakk Ø, Losnegard T, Skattebo Ø, Hegge AM, Tønnessen E, Kocbach J. Analysis of classical time-trial performance and technique-specific physiological determinants in elite female cross-country skiers. Front Physiol. 2016. 10.3389/fphys.2016.00326. 27536245 10.3389/fphys.2016.00326 PMC4971020

[139] Staib JL, Im J, Caldwell Z, Rundell KW. Cross-country ski racing performance predicted by aerobic and anaerobic double poling power. J Strength Cond Res. 2000;14(3):282–8.

[140] Zoppirolli C, Pellegrini B, Bortolan L, Schena F. Effects of short-term fatigue on biomechanical and physiological aspects of double poling in high-level cross-country skiers. Hum Mov Sci. 2016. 10.1016/j.humov.2016.02.003. 26904974 10.1016/j.humov.2016.02.003

[141] Martin-Rincon M, González-Henríquez JJ, Losa-Reyna J, Perez-Suarez I, Ponce-González JG, de La Calle-Herrero J, et al. Impact of data averaging strategies on VO(2max) assessment: Mathematical modelling and reliability. Scand J Med Sci Sports. 2019. 10.1111/sms.13495. 31173407 10.1111/sms.13495

[142] Decroix L, De Pauw K, Foster C, Meeusen R. Guidelines to classify female subject groups in sport-science research. Int J Sports Physiol Perform. 2016. 10.1123/ijspp.2015-0153. 26182438 10.1123/ijspp.2015-0153

[143] Coyle EF. Very intense exercise training is extremely potent and time efficient: a reminder. J Appl Physiol. 2005. 10.1152/japplphysiol.00215.2005. 15894535 10.1152/japplphysiol.00215.2005

[144] Faria EW, Parker DL, Faria IE. The science of cycling: physiology and training – Part 1. Sports Med. 2005. 10.2165/00007256-200535040-00002. 15831059 10.2165/00007256-200535040-00002

[145] Lucía A, Hoyos J, Santalla A, Pérez M, Chicharro JL. Kinetics of VO(2) in professional cyclists. Med Sci Sports Exerc. 2002. 10.1097/00005768-200202000-00021. 11828243 10.1097/00005768-200202000-00021

[146] Jeukendrup AE, Craig NP, Hawley JA. The bioenergetics of world-class cycling. J Sci Med Sport. 2000. 10.1016/s1440-2440(00)80008-0. 11235007 10.1016/s1440-2440(00)80008-0

[147] Formenti D, Trecroci A, Cavaggioni L, Caumo A, Alberti G. Heart rate response to a marathon cross-country skiing race: a case study. Sport Sci Health. 2015. 10.1007/s11332-014-0187-8.

[148] Hladnik J, Supej M, Vodičar J, Kerman B. The influence of boot longitudinal flexural stiffness on external mechanical work and running economy during skate roller-skiing: a case study. Proc Inst Mech Eng P J Sports Eng Technol. 2019. 10.1177/1754337119867546.

[149] Talsnes R, Moxnes EF, Nystad T, Sandbakk Ø. The return from underperformance to sustainable world-class level: a case study of a male cross-country skier. Front Physiol. 2023. 10.3389/fphys.2022.1089867. 36699686 10.3389/fphys.2022.1089867 PMC9870290

[150] Carlsson M, Calsson T, Hammarström D, Malm C, Tonkonogi M. Prediction of race performance of elite cross-country skiers by lean mass. Int J Sports Physiol Perform. 2014. 10.1123/ijspp.2013-0509. 24700141 10.1123/ijspp.2013-0509

[151] Jones TW, Lindblom HP, Karlsson Ø, Andersson EP, McGawley K. Anthropometric, physiological and performance developments in cross-country skiers. Med Sci Sports Exerc. 2021. 10.1249/mss.0000000000002739. 34649265 10.1249/MSS.0000000000002739

[152] Soli GS, Sandbakk Ø, McGawley K. Sex differences in performance and performance determining factors in the Olympic winter endurance sports. Sport Med Open. 2024. 10.1186/s40798-024-00792-8. 10.1186/s40798-024-00792-8 PMC11579258 39565528

[153] Ford P, De Ste Croix M, Lloyd R, Meyers R, Moosavi M, Oliver J, et al. The Long-Term Athlete Development model: physiological evidence and application. J Sports Sci. 2011. 10.1080/02640414.2010.536849. 21259156 10.1080/02640414.2010.536849

[154] Smith DJ. A framework for understanding the training process leading to elite performance. Sports Med. 2003. 10.2165/00007256-200333150-00003. 14719980 10.2165/00007256-200333150-00003

[155] Cowley ES, Olenick AA, McNulty KL, Ross EZ. “Invisible sportswomen”: the sex data gap in sport and exercise science research. Women Sport Phys Act J. 2021. 10.1123/wspaj.2021-0028.

[156] Smith ES, McKay AKA, Kuikman M, Ackerman KE, Harris R, Elliott-Sale KJ, et al. Auditing the representation of female versus male athletes in sports science and sports medicine research: evidence-based performance supplements. Nutrients. 2022. 10.3390/nu14050953. 35267928 10.3390/nu14050953 PMC8912470

[157] Bull FC, Al-Ansari SS, Biddle S, Borodulin K, Buman MP, Cardon G, et al. World Health Organization 2020 guidelines on physical activity and sedentary behaviour. Br J Sports Med. 2020. 10.1136/bjsports-2020-102955. 33239350 10.1136/bjsports-2020-102955 PMC7719906

